# On the benefits of self-taught learning for brain decoding

**DOI:** 10.1093/gigascience/giad029

**Published:** 2023-05-03

**Authors:** Elodie Germani, Elisa Fromont, Camille Maumet

**Affiliations:** Univ Rennes, Inria, CNRS, Inserm, IRISA UMR 6074, Empenn ERL U 1228, 35000 Rennes, France; Univ Rennes, IUF, Inria, CNRS, IRISA UMR 6074, 35000 Rennes, France; Univ Rennes, Inria, CNRS, Inserm, IRISA UMR 6074, Empenn ERL U 1228, 35000 Rennes, France

**Keywords:** self-taught learning, brain decoding, autoencoder, convolutional neural network, deep learning

## Abstract

**Context:**

We study the benefits of using a large public neuroimaging database composed of functional magnetic resonance imaging (fMRI) statistic maps, in a self-taught learning framework, for improving brain decoding on new tasks. First, we leverage the NeuroVault database to train, on a selection of relevant statistic maps, a convolutional autoencoder to reconstruct these maps. Then, we use this trained encoder to initialize a supervised convolutional neural network to classify tasks or cognitive processes of unseen statistic maps from large collections of the NeuroVault database.

**Results:**

We show that such a self-taught learning process always improves the performance of the classifiers, but the magnitude of the benefits strongly depends on the number of samples available both for pretraining and fine-tuning the models and on the complexity of the targeted downstream task.

**Conclusion:**

The pretrained model improves the classification performance and displays more generalizable features, less sensitive to individual differences.

## Introduction

In the past few years, deep learning (DL) approaches have achieved outstanding performance in the field of neuroimaging [1] due to their ability to model complex nonlinear relationships in the data. Functional magnetic resonance imaging (fMRI) data, a noninvasive neuroimaging technique in which brain activity is recorded during specific experimental protocols probing different mental processes and giving a big picture on cognition, are often used as input data to these models. These can be used for different purpose, such as disease diagnosis [2] or brain decoding (i.e., identifying stimuli and cognitive states from brain activities) [3], with a common goal: linking a target with highly variable patterns in the data and ignoring aspects of the data that are unrelated to the learning task. Researchers took advantage of the specific properties of fMRI data to build more and more sophisticated models [4–11].

However, training effective DL models using MRI data comes with many challenges [12]. Brain images differ from natural images that are typically used in the DL community on multiple aspects: they contain quantitative information (i.e., statistical values in our context of task fMRI that need to be considered differently during preprocessing steps such as standardization or during training with batch normalization), spatial localization is crucial information (i.e., the same activation in different regions of the brain leads to a completely different interpretation), and the dimensionality of medical images is much larger (i.e., an fMRI statistic map contains tens of thousands of dimensions). This means that a technique used for natural images may fail for medical images (see [13]).

While DL models have helped resolve important problems in subfields of brain imaging (such as the segmentation of anatomical datasets), their use in task fMRI is still limited [14]. Performance of DL models in task fMRI has been limited by the high dimensionality, lack of diversity, and low sample size of conventional datasets [15, 16]. In relation with the large number of trainable parameters in DL models [17], this makes it particularly difficult to build fair and generalizable DL models for fMRI. Indeed, fMRI datasets are typically composed of 3-dimensional (3D) volumes with hundred thousand dimensions (or voxels) for a rather small number of subjects (typically 10–100). The field also suffers from a large number of sources of variability in the data at the subject level (brain activity patterns differ across subjects), the acquisition level (fMRI scanners and protocols often vary between centers and studies), and the analysis level (different analysis pipelines lead to different brain patterns). In our case, brain decoding models should be robust to all these sources of variability [18].

This problem of small and uniform training sets is not limited to neuroimaging and is well known in the field of machine learning, where researchers extensively use deep transfer learning to improve classification and generalization performance of their models (see, e.g., [19]). This method consists in using the knowledge obtained from a model trained for a source task on a source dataset and applying it to a target task on a target dataset. Transfer learning proved its worth on natural images by using large, publicly available datasets [20] to pretrain DL models before fine-tuning them on smaller datasets of a related domain.

While the availability of large datasets has enabled solving difficult machine learning problems (such as classification), collecting similarly large datasets in brain imaging (including a diversity of participants and modalities) is especially challenging. In fact, in fMRI studies, the median sample size was still limited to *N* = 30 participants in 2015 [15]. Efforts for collecting large-scale datasets have arisen in the field in the past 10 years with, for instance, the Human Connectome Project (HCP) [21] or the UK Biobank [22] and give hope for an improvement of model performance. But these datasets are still much smaller than those that brought breakthroughs in computer vision and are often much less diverse [23].

To prevent overfitting and allow for generalizable statistical inference, neuroimaging researchers proposed methods to tackle this lack of training data [24–26]. For instance, Mensch & al. [27] built a decoding model using data gathered from 35 studies and thousands of individuals that cover various cognitive domains. Despite the good performance of the models, these can only be applied on restricted sets of studies, discriminating between few cognitive concepts. More annotated training data (e.g., using large public databases) would be required to map a wider set of cognitive processes.

Many studies were also made on inductive transfer learning with labeled source data as defined in [19] (e.g., source task and target task are different, as well as source domain and target domain) [28–30]. For instance, Thomas & al. [28] pretrained 2 DL classifiers on a large, public whole-brain fMRI dataset of the HCP, fine-tuned them, and evaluated their performance on another task on the same dataset and on a fully independent dataset. In another study, Gao & al. [29] used the ImageNet database [20], a large, public dataset containing naturalistic images from more than 1,000 classes, to pretrain a model and adapt it to classify tasks from 2-dimensional (2D) fMRI data. This database was also used in [31] for pretraining a 2D structural MRI classifier. In the same study, the Kinetics dataset [32] was also used to evaluate the transfer learning process with 3D images. In a recent work, Thomas & al. [33] used self-supervised learning frameworks to pretrain DL decoding models across a broad fMRI dataset, comprising many individuals, experimental domains, and acquisition sites. These studies show improved classification accuracies as well as quicker learning and less training data required.

However, labeled databases are not always available in neuroimaging, despite the growing effort in data sharing to build public databases [34], such as OpenNeuro for raw data [35] and NeuroVault for fMRI statistic maps [36]. The unconstrained annotations and the heterogeneity of tasks and studies make them difficult to use to pretrain a supervised DL model. To compensate this, weakly supervised learning techniques such as automatic labeling of data has proven its worth. For instance, Menuet & al. [37] enriched NeuroVault annotations using the Cognitive Atlas ontology [38] and used these labeled data to train a multitask decoding model that successfully decoded more than 50 classes of mental processes on a large test set.

A specific type of inductive transfer learning named *self-taught learning* [39, 40] showed strong empirical success in the field of machine learning. It does not require any labels as it consists in training models to autonomously learn latent representations of the data and using these to improve learning in a supervised setting. This approach is motivated by the observation that data from similar domains contain patterns that are similar to those of the target domain. By initializing the weights of a supervised classifier with the pretrained weights of an unsupervised model trained on many images, the aim is to improve the model performance by placing the parameters close to a local minimum of the loss function and by acting as a regularizer [41].

In the field of neuroimaging, latent representations have recently been used in a task-relevant autoencoding framework. In [42], an autoencoder was used with a classifier attached to the bottleneck layer on a small fMRI dataset. This model outperformed the classifier trained on raw input data by focusing on cleaner, task-relevant representations. This suggests that a low-level representation of fMRI data, learned for a reconstruction task, can be helpful in a classification task, as in a self-taught learning framework.

In this work, we propose to take advantage of NeuroVault—a large public neuroimaging database that was built collaboratively and therefore displays a good level of variability in terms of fMRI acquisition protocols, machines, sites, and analysis pipelines—in a self-taught learning framework. We pretrained an unsupervised DL model to learn a latent representation of fMRI statistic maps, and we fine-tuned this model to decode tasks or mental processes involved in several studies. In the first part, we leveraged the NeuroVault database to select the most relevant statistic maps and train a convolutional autoencoder (CAE) to reconstruct these maps. In the second part, we used the final weights of the encoder to initialize a supervised convolutional neural network (CNN) to classify the cognitive processes, tasks, or contrasts of unseen statistic maps from large collections of the NeuroVault database (an homogeneous collection of more than 18,000 statistic maps and an heterogeneous one with 6,500 maps). Our goal was to investigate how the use of a large and diverse database in a self-taught learning framework can be beneficial in the field of brain imaging for DL models.

## Material and Methods

The code produced to run the experiments and to create the figures and tables of this article is available in the Software Heritage public archive [43]. Derived data used by these notebooks are stored in Zenodo [44].

**Figure 1:**
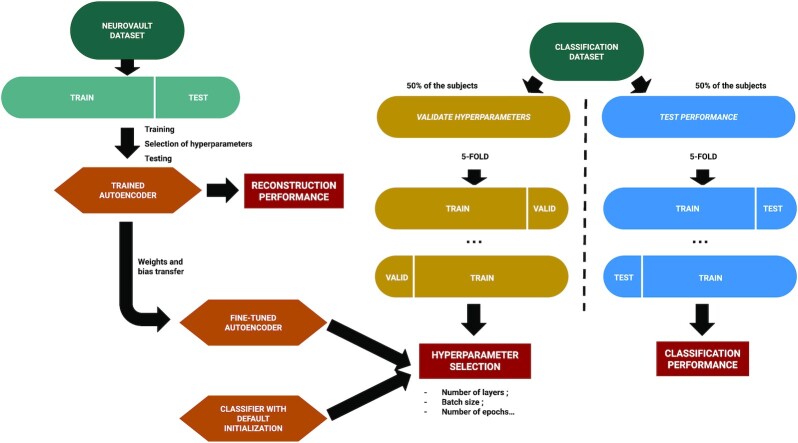
Flow diagram of the self-taught learning methodology. NeuroVault dataset is used to train a convolutional autoencoder (CAE). The encoder of this CAE is used to initialize a convolutional neural network (CNN) and to train it to classify other datasets. These classification datasets are split in 2 disjoints datasets: a “validation” one used to optimize hyperparameters and a “test” one to evaluate performance. In each one, a 5-fold cross-validation is performed.

Fig. 1 illustrates the overall process used to implement our self-taught learning framework: a CAE was first trained to reconstruct the maps of a large dataset extracted from NeuroVault. Then, the encoder part of the CAE was fine-tuned to answer a classification problem on another dataset (with labels). After hyperparameter optimization, performance of the pretrained classifier was compared to those of a classifier initialized with a default algorithm. Details regarding the datasets (NeuroVault dataset and classification datasets) can be found in the next subsection. The models of the CAEs and the CNNs are presented in section “Model architectures”. Further explanations on the workflow used to train the CAE and the CNN and to evaluate their performance are available in sections “CAE training” and “Classifier training”, respectively.

### Overview of the datasets

A summary of the different datasets can be found in Table 1. Details are given below.

**Table 1:** Overview of the datasets. For each dataset, number of statistic maps is presented, as well as the number of subjects, number of studies, and the type of labels (if available).

Dataset	Maps	Subjects	Studies	Labels
NeuroVault	28,532	—	—	—
HCP	18,070	787	1	Tasks (7)
				Contrasts (23)
BrainPedia	6,448	826	29	Cognitive
				processes (36)

#### NeuroVault dataset

NeuroVault [36] (RRID:SCR_003806) is a web-based repository for statistic maps, parcellations, and atlases produced by MRI and positron emission tomography studies. This is currently the largest public database of fMRI statistic maps. NeuroVault has its own public Application Programming Interface that provides a full access to all images (grouped by collections) and enables filtering of images or collections with associated metadata. At the time of the experiment (19 January 2022), a total of 461,461 images in 6,782 collections were available. Among the available metadata, some are mandatory and specified for all maps such as the modality (e.g., “fMRI-BOLD” for blood oxygen level dependent functional MRI; dMRI for diffusion MRI, etc.), the type of statistic (e.g., “T map” or “Z map”), or the cognitive paradigm (e.g., “working memory” or “motor fMRI task paradigm”), and others are optional and only available if additionally entered at the time of the upload.

From this large database, relevant maps were selected based on multiple criteria. First, we chose maps for which the modality was “fMRI-BOLD” to exclude other modalities such as structural or diffusion MRI. To get comparable maps, we set 3 additional inclusion criteria and selected maps (i) for which all required metadata were provided (“is_valid” to True), (ii) that were registered in MNI space (“not_mni” to False) to ensure that anatomical structures were located at the same coordinates in each map, and (iii) that were referenced as “T map” or “Z map” to exclude maps in which voxel values did not have the same meaning (e.g., *P* value maps, chi-squared maps). Among these, thresholded statistic maps were excluded.

We found that some maps in our initial dataset were wrongly referenced as T map or Z map. These misclassified maps were removed by filtering the “filename” column of the dataframe to exclude *SetA_mean SetB_mean* (AFNI contrast maps), *con* (SPM contrast maps), and *cope* (FSL contrast maps).

Using these criteria, a total of 28,532 statistic maps were selected from the NeuroVault database and constituted our “NeuroVault dataset.” Most of these maps were unlabeled (i.e., cognitive processes or tasks performed described as “None/Other”) or not labeled in a standardized way (i.e., use of terms that are specific for a study instead of generic terms, such as those defined in [38]; e.g., some maps were labeled as “word-picture matching task” for the cognitive paradigm, whereas others in which a similar task was performed were referenced as “working memory fMRI task paradigm,” which is a label that includes other specific tasks).

#### HCP dataset (NeuroVault Collection 4337)

NeuroVault collection 4337 [45] includes 18,070 *z*-statistic maps, for base contrasts (task vs. baseline), corresponding to 787 subjects of the HCP 900 release [46]. This collection was excluded from our pretraining dataset (see subsection  “NeuroVault dataset”) due to missing metadata (i.e., “is__valid” is false).

All maps in this collection were grouped together and referred to as the “HCP dataset” in the following. Multiple labels were entered for each map, including mental concepts (“cognitive_paradigm_cogatlas”), tasks (“task”), and contrasts (“contrast_definition”) (as defined in [38]). For each subject, 23 contrasts distributed in 7 tasks were available:

- Working memory: “0-back body,” “0-back face,” “0-back places,” “0-back tools,” “2-back body,” “2-back face,” “2-back places,” “2-back tools”
- Motors: “cue,” “left foot,” “left hand,” “right foot,” “right hand”, "tongue"
- Relational: “relational,” “match”
- Gambling: “punish,” “reward”
- Emotion: “faces,” “shapes”
- Language: “math,” “story”
- Social: “tom”

For more details on contrasts, tasks, and mental concepts of this study, see [46].

#### BrainPedia dataset (NeuroVault collection 1952)

NeuroVault collection 1952 [47], known as BrainPedia [48], contains fMRI statistic maps of about 30 fMRI studies from OpenNeuro [35], the HCP [46], and data acquired at Neurospin research center; together, they were chosen to map a wide set of cognitive functions.

This collection contains 6,573 statistic maps corresponding to 45 unique mental concepts derived from 19 subterms (e.g., “visual, right hand, faces” for maps associated with the task of watching an image of a face and responding to a working memory task). These images were previously used to build a multiclass decoding model [48], and labels corresponded to the mental concepts associated with the statistic map (e.g., “visual,” “language,” or “objects”). Here we excluded the 9 classes that had fewer than 30 samples each, leaving 6,448 images corresponding to 36 classes. These 6,448 images were grouped together and referred to as the “BrainPedia” dataset in the following.

### Preprocessing

All statistic maps included in this study were downloaded from different collections of NeuroVault and therefore were processed using different pipelines (see the original studies for more details [48, 46]). We resampled all maps to dimensions (48, 56, 48) using the MNI152 template available in Nilearn [49] (RRID:SCR_001362) as target image. A min–max normalization was also performed on all resampled maps to get statistical values between −1 and 1. Finally, the brain mask of the MNI152 template in Nilearn was used to exclude statistical values outside the brain in all statistic maps.

### Model architectures

**Figure 2:**
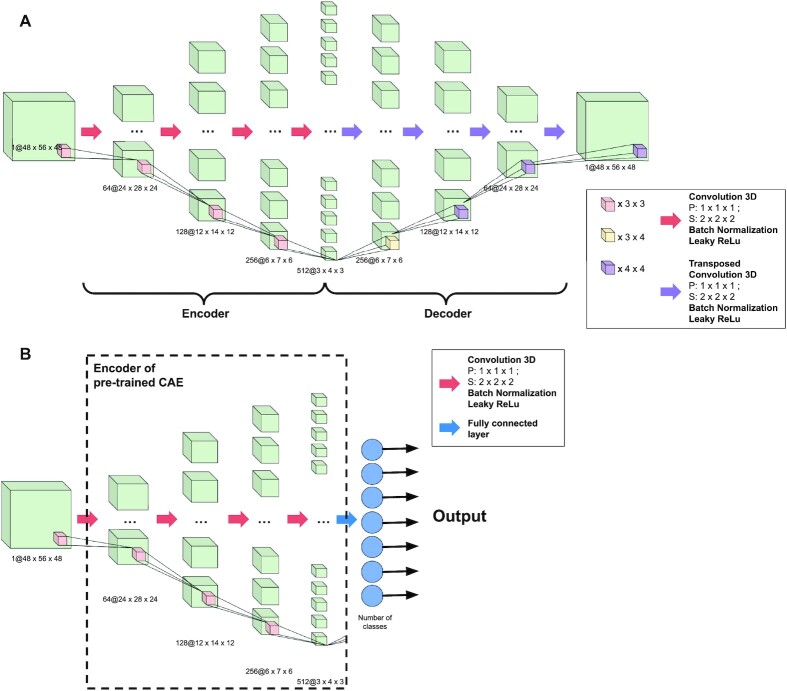
Schematic visualization of the architectures of the convolutional autoencoder (CAE) (A) and convolutional neural network (CNN) (B) with 4 layers. The CAE is composed of an encoder and a decoder with respectively 4 convolutional and transposed convolutional layers. The size of the latent space is 512 * 3 * 4 * 3. The CNN has the same architecture as the encoder of the CAE with a fully connected layer added at the end of the network with different numbers of output node depending on the dataset and the classification performed.

All models were implemented using PyTorch [50] v1.12.0 (RRID:SCR_018536) with CUDA [51] v10.2. For our model architectures, we chose to use 3D-convolutional feature extractors that take into account the 3 spatial dimensions of fMRI statistic maps. Schematic representations of the architectures are available in Fig. 2 and Supplementary Fig. S1, available at [52].

#### CAE

The base architecture of our CAE was inspired from [26]. Two architectures were derived from this base: a 4-layer and a 5-layer architecture, respectively corresponding to the number of convolutional layers in each part of the CAE (encoder and decoder). In the 4-layer model, the encoder part consisted in four 3D convolutional layers with respectively 64, 128, 256, and 512 channels. Each layer had a kernel size of 3 × 3 × 3, a stride of 2 × 2 × 2, and a padding of 1 × 1 × 1. The 3D batch normalization layers [53] followed each convolutional layer with respectively 64, 128, 256, and 512 channels, and a leaky rectified linear unit (ReLU) activation function was used for all layers. The decoding part of the CAE was symmetric to the encoder, except that 3D transposed convolutional layers were used instead of classic convolutional layers. Transposing convolutions is a method to upsample an output using learnable parameters. It can be seen as an opposite process to classical convolutions. To keep the number of features symmetric at each layer’s output, the kernel size of the first layer was set to 4 × 3 × 4 and to 4 × 4 × 4 for all other transposed convolutional layers. Leaky ReLU activation function was also used for all layers except for the last one (i.e., the output one), for which a sigmoid function was used in order to obtain output values between −1 and 1. The latent space for this model was of size 512 × 3 × 4 × 3. A schematic representation of this architecture can be found in Fig. 2A.

In the 5-layer model, 1 convolutional layer was added at the beginning of the encoder with 32 channels and similar parameters as the other layers of the encoder. A transposed convolutional layer was also added at the end of the decoder with 32 channels. The kernel sizes in the decoder were also modified to maintain the feature map sizes: the first and second layers of the decoder had kernel sizes of 3 × 4 × 3 and 4 × 3 × 4, respectively. All other parameters, batch normalization layers, and activation functions were the same. The latent space for this model was of size 512 × 2 × 2 × 2. A schematic representation of this architecture can be found in Supplementary Fig. S1A, available at [52].

#### CNN

The 3D CNNs used for classification followed the architecture of the encoder part of the CAEs. In the same way as for the CAEs, 2 CNN architectures were derived. For each one, we took the corresponding architecture of the encoder (4 or 5 layers) and added a fully connected layer at the end. The number of nodes in this layer varied depending on the number of classes. A softmax activation function was used for this output layer. Visual representation of the CNNs are available in Fig. 2B and Supplementary Fig. S1B, available at [52].

### CAE training

To train our CAEs to reconstruct the statistic maps of the NeuroVault dataset, we used an Adam optimizer [54] with a learning rate of 1e-04 and all other parameters with default values. The loss function was the mean squared error (MSE: the squared L2 norm), which is the standard reconstruction loss.

#### Dataset split

NeuroVault dataset was randomly split in 2 subsets: training and test with respectively 80% and 20% of the maps. The training set (*N* = 22,772 maps) was used to train the CAE with the different architectures and the test set (*N* = 5,760 maps) to assess the performance of the different models (with different hyperparameters).

#### Architecture comparison

To limit the computational cost of our experiments, we fixed some of the hyperparameters of the CAE and only compared those that were of interest for the later experiments. Here, we use the term model “hyperparameters,” to distinguish with model “parameters,” to represent the values that cannot be learned during training but are set beforehand (e.g., the batch size or the number of hidden layers). Thus, a batch size of 32 and a learning rate of 1e-04 were chosen to train the CAE for a number of 200 epochs (i.e., values that are often used in experiments). The only hyperparameter for which different values were compared included the number of hidden layers of the model: 4 layers vs. 5 layers for each part (encoder/decoder) of the model.

#### Performance evaluation

To assess the performance of the CAEs, we estimated Pearson’s correlation coefficient between the reconstructed statistic map and the original statistic map. The correlation coefficient was computed using numpy version 1.21.2 (RRID:SCR_008633) [55]. The closer to 1 the correlation coefficient was, the stronger the relationship between the maps and the more accurate the reconstruction. Note that we did not use MSE in this context as its individual values (for each data point) were not easily interpreted.

### Classifier training

We trained 2 types of classifiers for all the experiments:

- the *classifier with default algorithm* initialized with the original algorithm from [56] (i.e., Kaiming Uniform algorithm for convolutional and fully connected layers with a parameter of $\sqrt{5}$) and
- the *classifier with pretrained CAE* initialized using the weights and bias of the convolutional layers of the CAE pretrained on the NeuroVault dataset.

The CNNs were trained using the Adam optimizer with a learning rate of 1e-04. We used the cross-entropy loss function for training the classifier. Both were implemented in PyTorch.

#### Dataset split

As described in Fig. 1 (on the right), the classification datasets were split in 2 disjoint subsets: the *validation dataset* used to optimize the hyperparameters and the *test dataset* used to test the performance. Each subset contained 50% of the subjects of the overall dataset with no overlap to avoid any data leakage (see [57, 58]).

For each experiment, the validation and test datasets were then split into 5 folds for cross-validation. Subjects were randomly sampled in each fold in order to ensure that there was no overlap of subjects across folds. The identifiers of the subjects included in the different folds were saved for reproducibility. More details on the methods used to perform the 5-fold split for each dataset are specified in section “Benefits of self-taught learning and impact of different factors”.

#### Evaluation of performance

The performance of each model was measured using several metrics: accuracy (Acc), precision (P), recall (R), and F1-macro score (F1). All metrics were implemented using scikit-learn [49] with default parameters, except for F1-score, for which the “average” parameter was specified with “macro” to deal with multiclass classification.

To evaluate the performance of a model, all metrics were averaged among the 5 folds of cross-validation, and standard error of the mean was computed.

To compare the final performance of models with default initialization versus fine-tuned weights, we used paired 1-tailed 2-sample *t*-tests between the performance values (accuracy or F1-score) of the 5 models trained during cross-validation. The *t*-statistic and *P* value were provided, and a value of 0.05 was used for the *P* value significance threshold.

#### Hyperparameter optimization

To select the best hyperparameters for each dataset and each type of initialization, we evaluated the performance of each model by performing a 5-fold cross-validation on the validation dataset.

For each type of classifier (i.e., initialized with default algorithm vs. pretrained), we refined and optimized the hyperparameters using the largest datasets (Large BrainPedia and HCP). However, the large amount of training data made it computationally extremely costly to perform a full grid search. We therefore limited our research to predefined values of batch sizes (32 or 64), number of epochs (200 or 500), and model architectures (4 layers or 5 layers). All batch sizes, number of epochs, and architectures were tested for each type of classifier and each dataset. We did not perform any optimization on the learning rate to limit the computational cost of our experiments. Every model was trained using a learning rate of 1e-04.

We selected the best set of hyperparameters based on the performance of the corresponding model in terms of accuracy and F1-score, averaged across folds.

### Benefits of self-taught learning and impact of different factors

To investigate the benefits of self-taught learning for neuroimaging data, different brain decoding experiments were studied. For all, after optimizing the hyperparameters of the 2 models (i.e., the model with default initialization or with pretrained CAE and fine-tuned weights), we assessed the performance of these optimized models on the test dataset using a 5-fold cross-validation.

#### Homogeneous dataset (single study)

**Figure 3:**
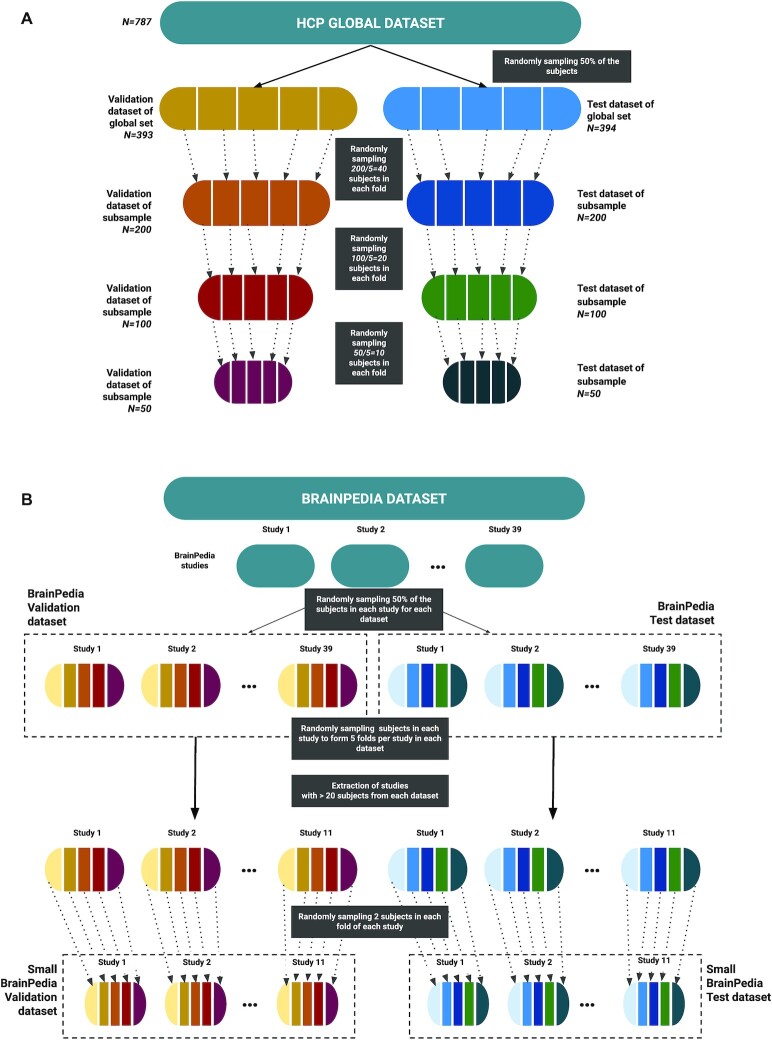
Overview of the process used to split the datasets for cross-validation. (A) The method performed for the HCP dataset and its subsamples. (B) Method used for BrainPedia and Small BrainPedia datasets. In both cases, the global dataset is first split into 2 subdatasets: “validation” and “test” with respectively 50% of the subjects, and then each subdataset is divided into 5 folds for cross-validation.

The HCP dataset was used to compare the performance of the models for the task of decoding on a homogeneous dataset (i.e., from a single study). We studied the impact of 2 factors on the classification: sample size and number of target classes. For sample size, subsets of the global HCP dataset were created with different number of subjects: *N* = 50, 100, and 200, with each smaller subset being a subset of the immediately larger one. To create these subsets, we first split the global HCP test dataset into 5 folds, with different subjects in each fold. In each of these 5 folds, we randomly sampled 200/5 = 40 subjects and obtained 5 subfolds that together composed the smaller subset of 200 subjects. This process was repeated for subsamples *N* = 100 and 50 by sampling from their superset. This ensured that the 5 models trained on different combinations of the 4 folds of a smaller subset could be tested on the remaining fold of the global test dataset with no overlap between the training and test data. The process is illustrated in Fig. 3A.

In the end, we obtained 4 datasets with respectively *N* = 50, 100, and 200 subjects in addition to the global dataset with all subjects (*N* = 393). These datasets respectively contained 1,150, 2,300, 4,590, and 9,017 statistic maps in the test subset and 1,150, 2,300, 4,591, and 9,053 in the validation subset (note: some contrasts were missing for part of subjects). Since we use a 5-fold validation scheme, the models were trained on approximately 80% of the statistic maps in the corresponding subset (i.e., validation for hyperparameter optimization and test for performance evaluation).

Three types of classification were investigated: first, the “contrast classification,” which consisted in identifying the contrast associated with a statistic map (23 different contrasts); second, the “task classification,” which consisted in identifying the task associated with a statistic map (7 different tasks, with multiple contrasts per task); and third, the “1-contrast task classification.” This time, we selected a single contrast per task and classified the tasks (7 different tasks, with 1 contrast per task). The selected contrasts were “2-back places,” “faces,” “punish,” “relational,” “right hand,” “story,” and “tom,” respectively for the tasks “working memory,” “emotion,” “gambling,” “relational,” “motor,” “language,” and “social.” We selected these contrasts similarly to what was done in [8], in which the HCP dataset was used in a decoding model. For each task, the contrast that showed a greater association with the task had priority over the other (e.g., “punish” for the “gambling” task). For “working memory” and “motor” tasks, which contained more than 1 task condition, the authors of [8] randomly chose one (“2-back body” for working memory and “right hand” for motor). The dataset used for this third type of classification was thus smaller than the others (only 1 map per task per subject). For this classification task, the number of statistic maps was respectively 300, 598, 1,198, and 2,355 for *N* = 50, 100, and 200 and for the global dataset.

#### Heterogeneous dataset (multiple studies)

To study the benefits of self-taught learning on a heterogeneous dataset (i.e., from multiple studies), we used BrainPedia. For these experiments, we focused on the classification of mental concepts (as available in NeuroVault metadata). Fig. 3B illustrates the process used to split this dataset. To perform the split while maintaining the heterogeneity in each fold, we randomly sampled 50% of the subjects of each study to form the “validation” and “test” datasets of BrainPedia (see Fig. 3B). Then, each dataset, each study was split into 5 folds, and the *n*th folds of the different studies were combined to form the *n*th fold of the dataset. Validation and test datasets included *N* = 428 subjects and were respectively composed of 3,179 and 3,269 statistic maps.

We also studied the impact of sample size in the presence of heterogeneity by extracting smaller datasets. Among the 29 studies of the BrainPedia dataset, we only kept those that were composed of more than 20 subjects. In these remaining studies, already split into 5 folds in BrainPedia validation and test subdatasets, 2 subjects were randomly drawn per fold per study per subdataset to obtain 10 subjects per study per subdataset. Like above, the *n*th folds of the different studies were combined to form the *n*th fold of each subdataset of the “Small BrainPedia” dataset. In the end, this smaller dataset was composed of 1,844 maps, divided in 30 classes, from 11 studies and 220 subjects. This dataset was also split into test and validation subsets with 50% of the subjects in each (*N* = 110). The test and validation subsets were thus composed respectively of 917 and 927 maps.

### Explainability

#### Exploring feature maps to understand the generalizability across subjects

To investigate the reasons for the difference in performance between the pretrained and default models, we visualized and analyzed the feature maps of the different convolutional layers of the model. Visualizing these features was useful to better understand how each model made its predictions. Differences in the observed features can help understand differences in terms of performance.

With a generalizable classifier, we hypothesized that features of different subjects from the same class should be similar (and therefore not be impacted by individual differences). To study this, we computed for each classifier, each layer, and each class the correlations between the feature maps for all pairs of subjects. A high mean correlation highlighted a higher similarity between the feature maps extracted by this layer for a classifier and thus a higher generalizability.

#### Investigating the contribution of each layer to the overall performance

We explored which pretrained layer had the strongest impact on the classification performance. This could be made at 2 stages: before and during training.

Before training, we only transferred the weights of some parts of the CAE. In particular, we kept the weights of the last convolutional layers with a default initialization and initialized the first layers with the weights of the pretrained CAE. Multiple configurations were explored: transferring only the weights of the first one up to the first 4 convolutional layers.

During training, we froze some layers of the model initialized with the weights of the pretrained CAE, that is, some layers (the first ones) were not fine-tuned. Multiple types of freezing were tested: freezing of the first 2 to the first 5 convolutional layers.

## Results

### AutoEncoder performance

**Figure 4:**
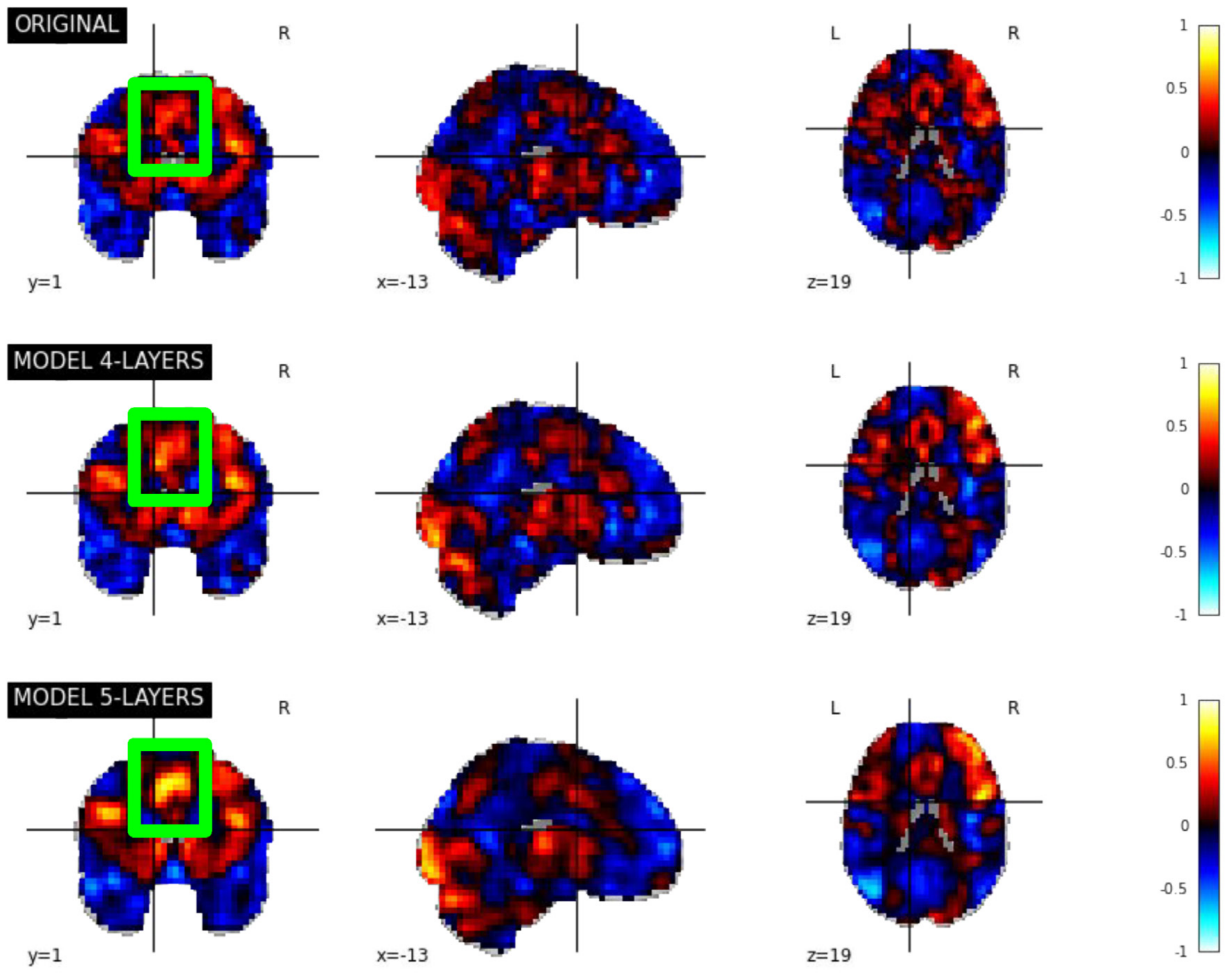
Original version and reconstruction of a randomly drawn statistic map of the NeuroVault test dataset (image ID: 109) with the 2 CAEs (4 layers and 5 layers). The green square corresponds to a highlighted part of the map for which reconstruction performance is better using the 5-layer architecture.

Reconstruction performance of the CAEs is presented in Table 2. When comparing the 2 CAE architectures (4 layers vs. 5 layers) trained on the NeuroVault dataset, the mean correlations between original and reconstructed maps were better for the 4-layer architecture (86.9% vs. 77.8%). These results suggest that the reconstruction capabilities of the CAEs are dependent on the model architecture and the size of the latent space. Fig. 4 shows the reconstruction of a statistic map randomly drawn from the NeuroVault test dataset with the 2 CAE architectures. With the 4-layer architecture, details of the map were better reconstructed than with the 5-layer architecture (see the green square on the map). This was due to the level of compression of the data that was higher in the 5-layer CAE and that learned only the most useful features with less emphasis in learning specific details. Both models were used as the pretrained model for classification to see if the benefits of the CAEs were related to their reconstruction performance.

**Table 2:** Reconstruction performance of the CAE depending on model architecture and training set. Values are the mean Pearson’s correlation coefficients (standard error of the mean).

Model	4 layers	5 layers
	*Latent space 18,432*	*Latent space 4,096*
Correlation	86.9	77.8
(*standard error*)	(*0.18*)	(*0.23*)

### Hyperparameter optimization for classifiers

The best hyperparameters and corresponding performance can be found in Table 3.

**Table 3:** Hyperparameters chosen for each dataset and corresponding performance of the classifier on the validation set of the dataset

Dataset	Initialization	Model	Epochs	Batch	Average accuracy (%)	Average F1 (%)
					(*standard error*)	(*standard error*)
HCP	Default algorithm	4 layers	500	32	90.8 (*1.5*)	90.8 (*1.6*)
	Pretrained CAE	5 layers	200	64	91.8 (*0.9*)	91.8 (*0.9*)
BrainPedia	Default algorithm	5 layers	500	64	67.1 (*1.7*)	61.0 (*1.6*)
	Pretrained CAE	5 layers	200	64	73.8 (*2.7*)	70.0 (*2.3*)

#### Choice of hyperparameters for HCP dataset

Performance of the different models trained with the different hyperparameters can be found in Supplementary Table S1, available at [52]. For the default algorithm initialization, the best model had 4 layers and was trained with a batch size of 32 for 500 epochs. This model achieved an accuracy of 90.8% on average of the 5 folds of cross-validation. For the pretrained CAE initialization, the best model had 5 layers and was trained with a batch size of 64 for 200 epochs (average accuracy of 91.8%). The best hyperparameters for each type of initialization (default and pretrained) were used in all subsequent experiments.

#### Choice of hyperparameters for BrainPedia dataset

Results for all sets of hyperparameters are available in Supplementary Table S2, available at [52]. For the default algorithm initialization, the model that achieved the best performance had 5 layers and a batch size of 64 for 500 epochs. This model classified the BrainPedia dataset with an average accuracy of 67.1% and an average F1-score of 61%. The performance of the pretrained CAE was the best using a 5-layer architecture, a batch size of 64, and a training time of 200 epochs.

### Benefits of self-taught learning on a homogeneous dataset

Table 4 summarizes the results for the different classification experiments on the HCP datasets.

**Table 4:** Classification performance on HCP datasets of models initialized with default algorithm vs. with the weights of the pretrained CAE. Mean accuracies and standard errors of the means among the 5 folds of cross-validation are shown. Paired 2-sample *t*-tests are performed between the accuracies of the 5 models obtained with cross-validation for each type of initialization. DA, default algorithm initialization; PT, pretraining initialization.

Subjects	50		100		200		Global (393)	
Maps	1,150		2,300		4,590		9,017	
Init.	DA	PT	DA	PT	DA	PT	DA	PT
**Contrast classification** (*23 classes*)								
Mean accuracy (%)	83.6	87.0	86.8	89.9	88.6	90.2	90.9	92.4
(standard error)	(0.61)	(0.51)	(0.69)	(0.34)	(0.84)	(1.46)	(0.38)	(0.44)
Paired *t*-test (*4 dof*)		−**11.52**		−**4.77**		−1.42		−**4.74**
*P* value		**0.0003**		**0.009**		*0.23*		**0.009**
**Task classification** (*7 classes, multiple contrasts per class*)								
Mean accuracy (%)	96.6	97.3	95.4	98.0	97.9	98.5	98.4	99.0
(standard error)	(0.47)	(0.43)	(1.49)	(0.25)	(0.44)	(0.16)	(0.17)	(0.13)
Paired *t*-test (*4 dof*)		−*3.57*		−1.4		−1.5		*−5.65*
*P* value		**0.02**		*0.2*		*0.2*		**0.005**
**One constrast task classification** (*7 classes, 1 contrast per class*)								
Mean accuracy (%)	97.9	99.1	98.9	99.4	99.3	99.6	99.4	99.6
(standard error)	(0.3)	(0.3)	(0.17)	(0.25)	(0.2)	(0.2)	(0.2)	(0.14)
Paired *t*-test (4 *dof*)		−**4.17**		−**3.32**		−2.33		−2.06
*P* value		**0.01**		**0.03**		*0.08*		*0.1*

#### Impact of the sample size

**Figure 5:**
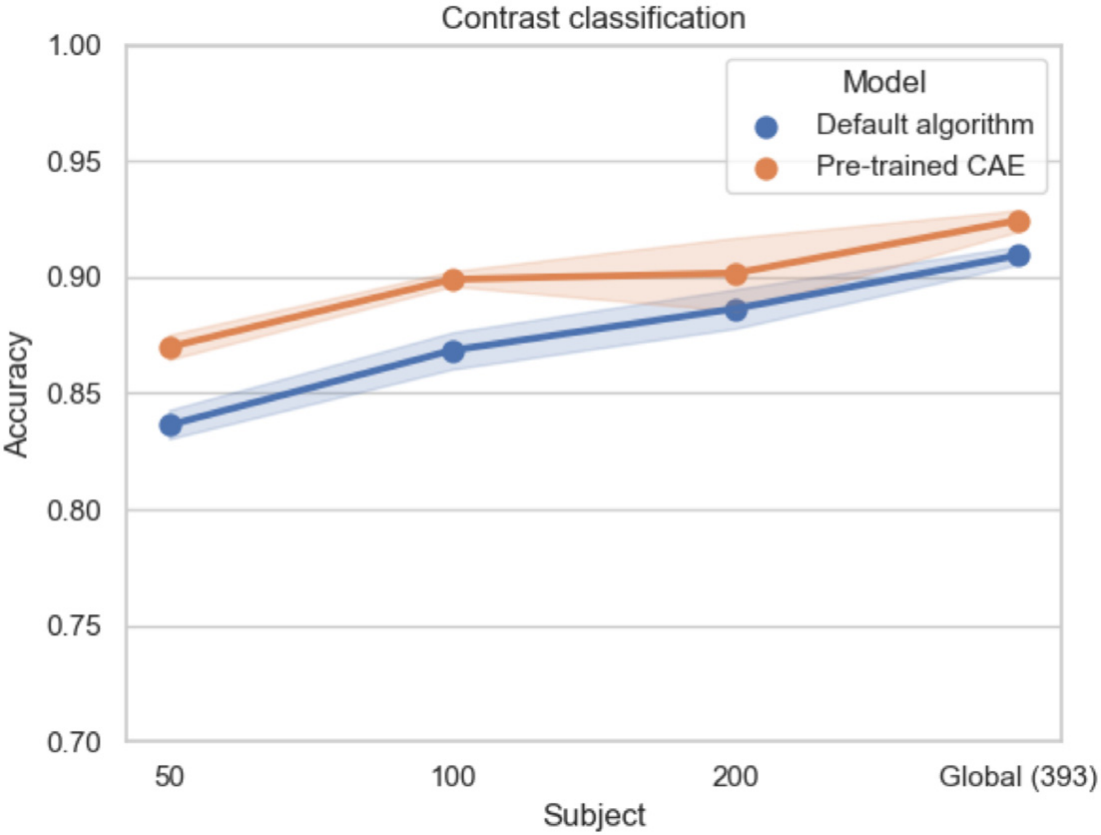
Mean accuracies and standard errors of the mean on contrast classification with the HCP dataset for the models initialized with default algorithm (blue) and pretrained CAE (orange). Pretraining improves contrast classification performance for small sample sizes and at a lower level of improvement, also for large sample sizes.

For all classification experiments, the size of the training set (in terms of number of subjects) had a strong impact on the benefits of self-taught learning. With 50 subjects, the performance of the pretrained CAE outperformed the performance of the classifier initialized with the default algorithm in all our experiments (improvements of 0.7% to 3.4% in mean accuracies). These improvements were always significant (*P* < 0.05). When sample size increased, this improvement was reduced and was sometimes not significant. If we focus on contrast classification (Fig. 5), which was the hardest classification task between the 3 presented here due to the higher number of classes, the difference between the performance of the 2 classifiers decreased with sample size (mean accuracies of 88.6% and 90.2%, respectively, for default initialization and pretrained model for *N* = 200, which corresponded to an improvement of 1.6% compared to almost 3% for *N* = 100). For *N* = 200, the difference of performance was not significant, probably due to the presence of an outlier value in the accuracies of the pretrained CAE. Indeed, accuracies of the pretrained CAE model were superior to the ones of the default model, except for the pretrained model tested on the third fold of cross-validation, which was lower. This value was also significantly lower than those of models tested on other folds of cross-validation (see Supplementary Table S3, available at [52]).

#### Impact of the target classification task

**Figure 6:**
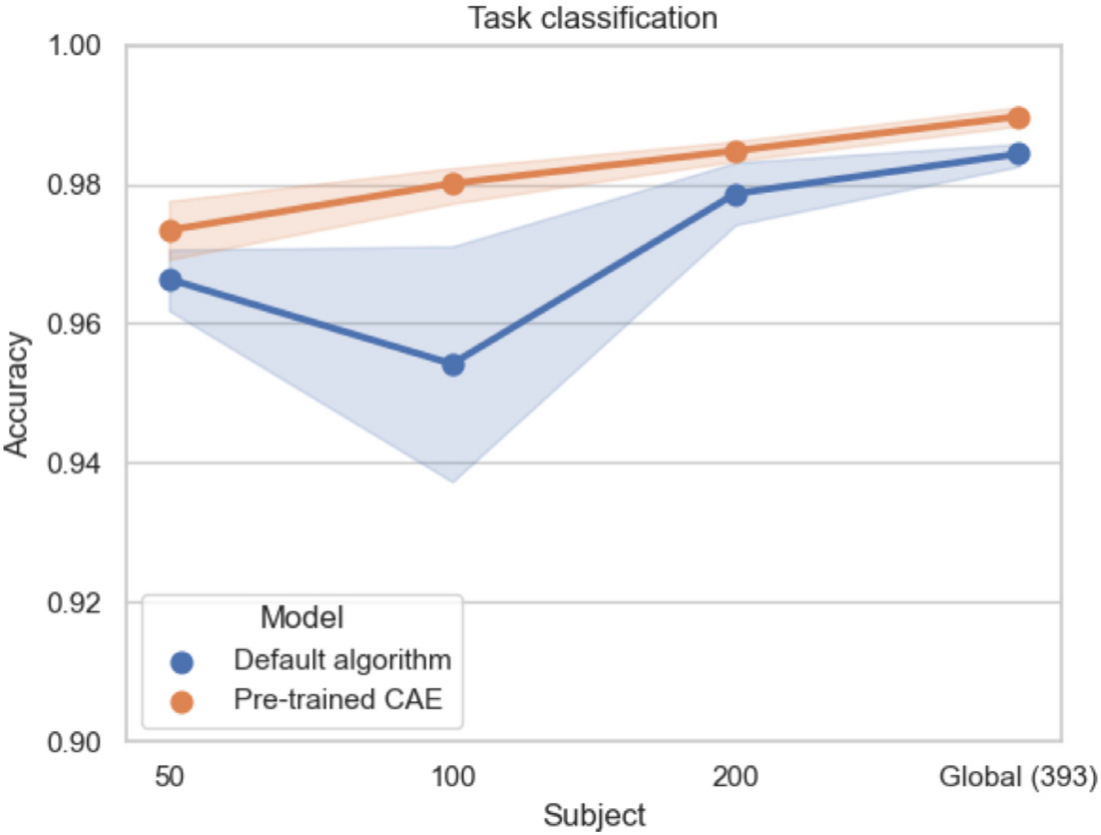
Mean accuracies and standard errors of the mean on task classification with the HCP dataset for the models initialized with default algorithm (blue) and pretrained CAE (orange). Pretraining improves task classification performance for all sample sizes, but sample sizes did not have a huge influence on the level of improvement.

**Figure 7:**
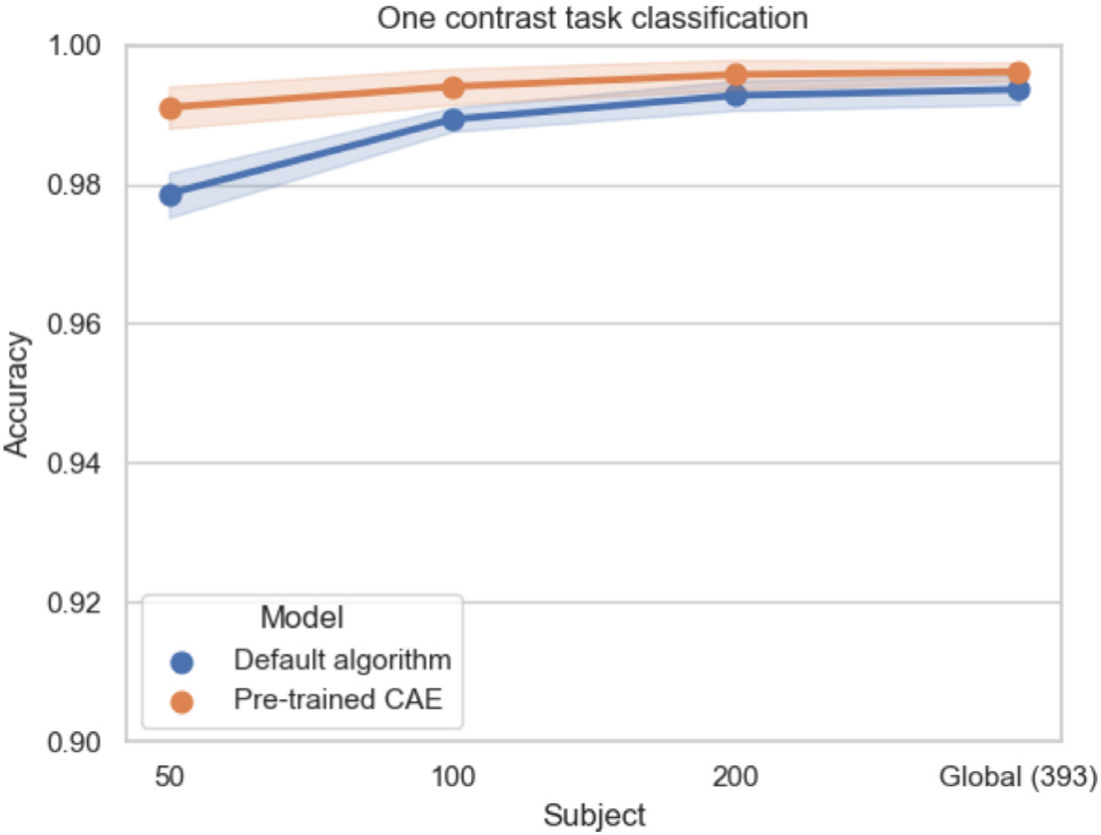
Mean accuracies and standard errors of the mean on 1-contrast task classification with the HCP dataset for the models initialized with default algorithm (blue) and pretrained CAE (orange). Pretraining does not always improve 1-contrast task classification performance: for large sample sizes, pretraining and default initialization give very similar results.

For simpler classification experiments (i.e., with less classes to separate), pretraining was not always useful. In these experiments, performance was already nearly perfect (accuracies close to 1) and therefore difficult to improve. For large sample sizes (*N* > 100), performance was close (difference between mean accuracies lower than 0.6%) between models initialized with default algorithm and pretrained models (see Figs. 6 and 7). However, for smaller sample sizes (*N* = 50), pretraining improved classification—similarly to what had been shown for more complex tasks—with accuracies of the pretrained models higher than default models of 0.7% and 1.2% for task classification and 1-contrast task classification, respectively. These results suggest that pretraining can be beneficial when studying difficult classification problems such as those with few training samples or complex classification tasks.

### Benefits of self-taught learning on a heterogeneous dataset

**Figure 8:**
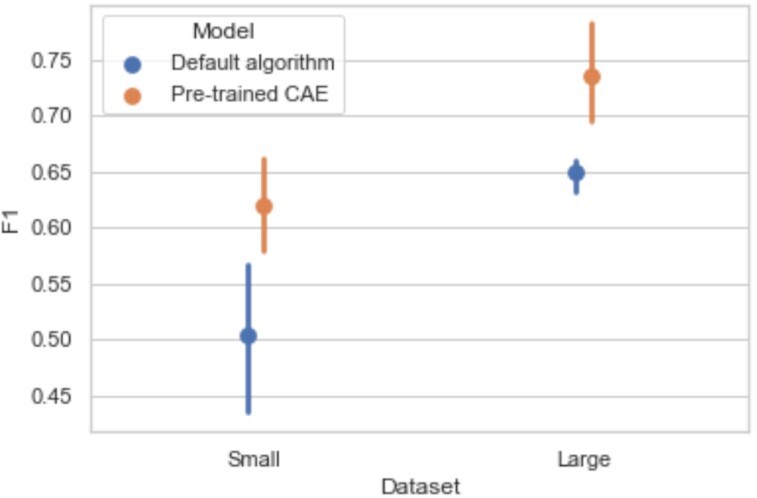
Mean F1-scores and standard errors of the mean of the classification of mental concepts on BrainPedia datasets (small and large) for the models initialized with default algorithm (blue) and pretrained CAE (orange). Pretraining improves classification performance, in particular for the small dataset.

Table 5 summarizes the results for the classification of mental concepts on the small and the large BrainPedia datasets. These results are illustrated in Fig. 8.

**Table 5:** Classification performance on BrainPedia datasets of models initialized with default algorithm vs. with the weights of a pretrained CAE. DA, default algorithm initialization; PT, pretraining initialization.

Dataset	Small BrainPedia		BrainPedia	
Init.	DA	PT	DA	PT
**Mean accuracy (%)**	56.8	64.5	67.1	74.2
(standard error)	(1.5)	(2.1)	(0.9)	(2.3)
Paired *t*-test (*4 dof*)		−**8.72**		−**3.43**
*P* value		**0.001**		**0.02**
**Mean F1-score (%)**	50.5	62.0	64.9	73.6
(standard error)	(3.5)	(2.1)	(0.8)	(2.2)
Paired *t*-test (*4 dof*)		−**4.89**		−**2.89**
*P* value		**0.008**		**0.04**

On a the small BrainPedia dataset, pretraining improved the performance of the classifier. When looking at the mean accuracies, respectively 56.8% and 64.5% for the classifier initialized with the default algorithm and the pretrained classifier, the difference was high (almost 8% of improvement). But in this case, the F1-score was a better metric to assess the performance. Indeed, this metric focuses more on classification errors and is a better indicator of performance when classes are imbalanced, which was the case in this dataset in which some classes were more represented than others (e.g., in the small BrainPedia training set, 205 maps corresponded to the class “visual words, language, visual,” whereas only 19 are in the class “left foot, visual”). When focusing on this metric, the pretrained classifier performance was markedly higher than the ones of the classifier with default initialization (11.5% of improvement in mean F1-score). Performance (accuracies and F1-scores) was both significantly improved with the pretrained model compared to the default one (*P* < 0.05).

On the global BrainPedia dataset, performance also increased with pretraining. Mean accuracy and F1-score were higher for the the pretrained model (F1-score of 73.6% against 64.9% for the model with default initialization) even if the sample size of the dataset was higher and more classes were represented. Indeed, the classification task was also more complex for this dataset since data were separated into 36 classes instead of 30 for Small BrainPedia due to the presence of maps from other studies in the dataset.

### How do we explain these benefits?

#### Features

To better understand the behavior of each model—in particular, on what features they based their predictions on—we visualized the mean features across subjects of each layer of the pretrained, default models and baseline CAE for each class label (i.e., contrast). Specifically, we studied the mean feature maps obtained across subjects in the test set (fold 1) of the *N* = 50 sample of the HCP dataset for different contrasts. This configuration was chosen due to the large difference between performance of default and pretrained models on this classification task. Our main interest was to see if the model would focus on general patterns of activation or more individual features. We focused on the contrasts that led to the most difficult classification tasks (i.e., had the lowest per-class accuracy [less than 80%]). Per-class accuracy for selected contrasts are shown in Table 6 and for all contrasts in Supplementary Table S7, available at [52]. Eight contrasts were selected: “Working memory”: “0-back body,” “0-back places,” “0-back tools,” “2-back body,” “2-back tools,” “Gambling: punish,” “Gambling: reward,” and “Relational: relational” and among these 8 contrasts, 7 (all except “2-back body”) had a better per-class accuracy with the pretrained CAE, see “Benefits of self-taught learning and impact of different factors”.

**Table 6:** Per-class accuracies for classification of contrasts with HCP dataset sample *N* = 50 for DA (default algorithm) and PT (pretrained CAE). Only lowest per-class accuracy (<80%) is shown in. For other per-class accuracy, please refer to Supplementary Table S7, available at [52].

Contrast	Per-class accuracy	
	DA	PT
**WM: 0BK BODY**	57.7	60.3
**WM: 0BK PLACE**	70.5	79.5
**WM: 0BK TOOL**	57.8	66.7
**WM: 2BK BODY**	74.3	73.1
**WM: 2BK TOOL**	47.4	60.3
**GAMBLING: PUNISH**	55.1	67.9
**RELATIONAL**	58.9	75.6
**GAMBLING: REWARD**	57.7	66.7

**Figure 9:**
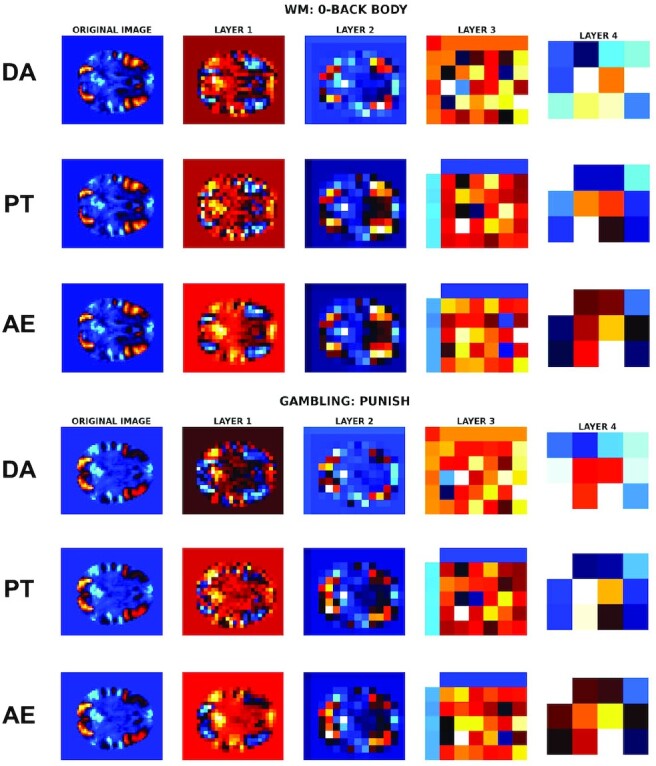
Original mean statistic maps (column 1) and mean feature maps across subjects of fold 1 of the test dataset of HCP 50 for the first 4 convolutional layers of each model (columns 2–5): CNN with default algorithm initialization (DA), pretrained CNN (PT), and CAE, for 2 of the 8 selected contrasts (WM: 0-back body and Gambling: punish).

Fig. 9 shows the mean feature maps for two of the selected contrasts and for the first 4 convolutional layers of the models: CNN with default initialization, pretrained CNN, and CAE. The mean feature maps of all the selected contrasts and layers are displayed in Supplementary Fig. S2, available at [52]. The first convolutional layer features (column 2 of Fig. 9) were similar across models but different between the contrasts: see, for instance, the activation patterns of contrasts WM: “0-back body” and “Gambling: punish,” which were localized in the same areas but had different shapes. These were high-level features: brain shape and main activation patterns. However, the second convolutional layer (third column) seemed to learn more important features for classification. The shape of the brain was still visible, but patterns of activation were more blurry, as if they were lower-resolution representations of the original statistic maps. However, features started to be different between models at this layer with some modifications of the shape of the main activation patterns between the default model (first row of each contrast) vs. the pretrained model and the CAE (second and third lines). The same observation was made for the third convolutional layer (fourth column), which began to learn deeper representations. Due to the size of the features (6 * 7 * 6), the brain shape and activation patterns were not visible, and these features were thus less interpretable and required a quantitative analysis.

**Figure 10:**
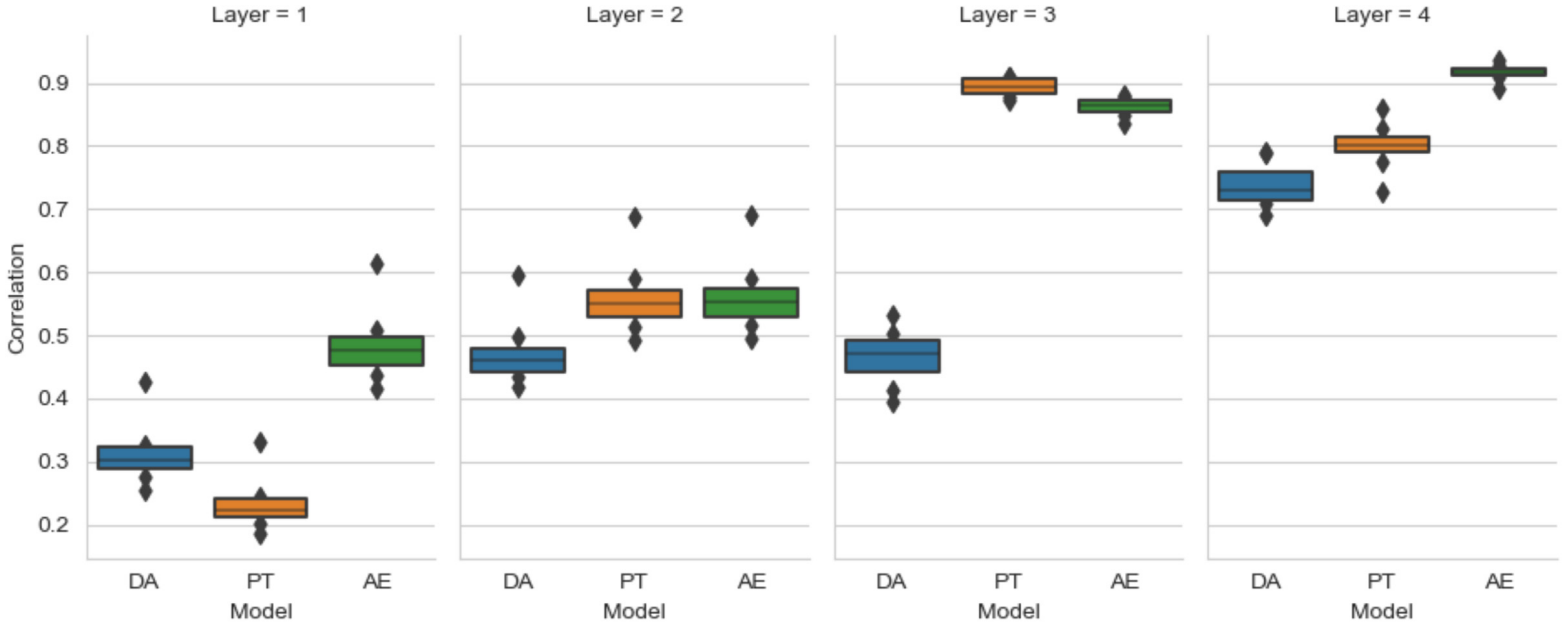
Boxplots of mean correlations between the feature maps of different subjects for the 8 selected contrasts (“Working memory”: “0-back body,” “0-back places,” “0-back tools,” “2-back body,” “2-back tools,” “Gambling: punish,” “Gambling: reward,” and “Relational: relational”) for different models at layers 1, 2, 3 and 4. DA, default algorithm initialization; PT, pretraining initialization; AE: baseline autoencoder. For layers 3 and 4, pretrained CNN and baseline CAE show larger correlation between subjects than default CNN, meaning a lower attention to individual variabilities.

Mean correlations between the feature maps of the same contrast were computed for each pair of subjects. A high mean correlation indicates a higher similarity between the feature maps produced in a given layer of a neural network and thus potentially a higher generalization power since the feature maps are less different between subjects and thus less sensitive to individual variations. Fig. 10 shows the mean correlations for the 8 selected contrasts and for the first 4 convolutional layers of the models (different values represent different contrasts). For layers 1 and 2, mean correlations were low (<60%) and not very different between the models even if the pretrained CNN seemed to account more about individual differences than the default model and baseline CAE. The main change was visible at layer 3, where there was an important difference (more than 30% for every contrast) between the mean correlation between the features learned by the default CNN and the pretrained one. The features of this layer seemed more similar between different subjects and more generalizable across subjects for the pretrained model (mean correlations >80% for all contrasts) than for the default model for which the mean correlations were lower than 50% for every contrast. Correlations started to converge for the fourth layer but were still lower for the default model.

#### What layers benefit the most from weight transfer from the CAE?

To explore the impact of each layer and the benefits of the baseline weights of the CAE, we tried several experiments with different numbers of frozen layers and several weight transfer configurations: transferring only the weights of the first convolutional layer to transferring the weights of the first 4 convolutional layers. Performance of the different models with different numbers of transferred layers is shown in Table 7. When only the weights of the first layer were transferred, classification performance was lower than with other configurations (82.7% of accuracy compared to more than 84% for at least 2 transferred layers). This suggests that features learned by the CAE at this layer were less important for classification. However, when increasing the number of transferred layers, performance started to grow and became closer to the accuracy obtained when transferring all layers (87%). This growth was quite constant, and there was no large improvement of performance when transferring the weights of a layer in particular, except when moving from transferring the first layer to the first 2 layers. Thus, pretraining the deeper layers of the model was beneficial to improve classification performance, probably because of the ability of these layers to extract more general features, less sensitive to individual variations, as we saw above. Transferring the weights of the last convolutional layer (fifth) was, however, not very impactful, and performance of model with 4 transferred layers was very close to the ones of the fully pretrained model (86.6% vs. 87.0%). We suppose that this layer was important to extract task-related features that were different from the ones learned by the CAE, explaining the limited impacts of transferring the CAE weights.

**Table 7:** Classification performance (mean accuracy and standard error, in %) of pretrained models with different numbers of transferred layers on classification of contrasts for HCP dataset sample, *N* = 50

Number of transferred layers	Mean classification accuracy (standard error) (%)
**0 (Default initialization)**	83.6 (0.61)
**1**	82.67 (0.45)
**2**	84.79 (0.52)
**3**	85.51 (0.8)
**4**	86.6 (0.4)
**Full pretrained model**	87.0 (0.51)

#### Faster fine-tuning: what happens if we freeze some layers?

Table 8 shows the results of the different experiments with different numbers of frozen layers. When we froze the first convolutional layers (from 2 to 4 frozen layers) on the pretrained model, the performance did not decrease. This suggests that the features extracted by the baseline autoencoder for these layers were general enough to perform a classification task with only 1 fine-tuned convolutional layer in addition to the dense layer. However, when freezing all convolutional layers of the model (5 layers), there was a large drop in terms of performance (86% to 80% of accuracy between freezing 2–4 layers vs. 5 layers), and this confirmed the observation made before on the difference between the features extracted by the fifth layer for reconstruction (CAE) and for classification (CNN). In conclusion, the first 4 convolutional layers of our model extracted more general features, whereas the last one extracted deeper and more specific features for classification.

**Table 8:** Classification performance (mean accuracy and standard error, in %) of pretrained models with different numbers of frozen layers on classification of contrasts for HCP dataset sample, *N* = 50

Nb. of frozen layers	Mean classification accuracy (standard error) (%)
**2**	86.7 (0.54)
**3**	86.82 (0.66)
**4**	86.1 (0.64)
**5**	80.42 (0.99)

## Discussion

### Summary of the results

In this work, we showed the benefits of self-taught learning with a large and variable database on the classification of 2 large public datasets with different sample sizes and classification tasks. In all cases, pretraining a classifier with an unsupervised task (in our case: reconstruction) was beneficial, but the level of improvement varied depending on the classification task and the size of the training dataset.

When sample sizes were small, pretraining always improved the classification performance, regardless of whether the dataset was homogeneous or heterogeneous and the complexity of the classification task. In medical imaging, where the dimensions of the data are often very large and few samples are typically available due to high financial and human costs, learning a good representation of the data can be very difficult [12, 59]. Unsupervised pretraining can thus be helpful by initializing the weights of the CNN to preserve the (brain) structure learned by the autoencoder and facilitate the learning process. However, when the sample size increases, benefits are less remarkable since the amount of available training data is probably sufficient to learn a good representation.

This observation can also be made for classification tasks. When trying to classify the data in a small number of classes, performance of the pretrained classifier was better but not with a high improvement of performance, even for small sample sizes (e.g., 100 subjects for task classification). But when trying to separate data into more classes, for a more fine-grained classification, the representation learned during the pretraining was beneficial.

Another benefit of self-taught learning we found was the reduction of the training time. Performance of the pretrained classifier was better even with less training epochs. This was the case for both dataset results, which were computed for 500 epochs for the default algorithm and 200 epochs for the pretrained model. This is in line with [60] in which researchers showed that the pretrained models remain in the same basin of the loss function when trained on new data, and since the weights are already initialized close to a good representation of data, less epochs are necessary to adapt this representation for classification.

Architectures of the models also had an impact on the benefits of self-taught learning. With both datasets, pretrained models performed better using the 5-layer architecture. This effect was studied by [41], who showed that, while unsupervised pretraining helps for deep networks with more layers, it appears to hurt for too small networks. The size of the latent space of the CAE, with 5 layers being almost 5 times smaller than the 4-layer one, suggests that only a small subset of features of the input is relevant for predicting the class label.

However, the classification accuracies of the pretrained models were not related to the reconstruction performance of the CAE since the 4-layer CAE reconstructs maps with better precision than the 5-layer CAE. This confirms that the features learned by the 4-layer CAE for reconstruction were not all useful for classification, and focusing on a smaller number of features (with 5 layers) facilitates the learning process.

This observation was confirmed by the large drop in performance when freezing the first fifth convolutional layers of the pretrained model and when transferring only part of the layers. Deeper pretrained layers had more impact on classification performance, meaning that the features extracted by these layers were different from those learned by layers initialized with the default algorithm. In particular, the third and fourth convolutional layers showed the best benefits when being transferred, due to the generalizability of the extracted features. This was not the case for the fifth layer, for which features need to be specific to the classification task.

The pretrained model improved the performance in terms of classification due its ability to focus on more generalizable features. By pretraining a model on a large variable dataset such as NeuroVault, we built a model that is less sensitive to the training data and less sensitive to individual differences, thus more generalizable and applicable to new subjects.

### Limitations and perspectives

Due to the high computational time required to train a model, we only compared 2 model architectures (4 and 5 layers). Indeed, training a CAE model can be very time-consuming, particularly in our case since we use a large training dataset (*N* = 22,772) and high-dimensional data (*k* = 48 * 56 * 48). With the 4-layer model, for 200 epochs, it took approximately 48 hours to train on 1 GPU. With parallel computing (use of 2 GPUs in parallel), we could hope to shorten this time to 24 hours, with the cost of using more computing resources. Other types of architectures with different number of fully connected or convolutional layers could have been tested to see the effect of other latent space sizes as it was done in [41].

The main limitation of our work is the classification experiments and datasets we chose. In fMRI, the number of possible labels and, thus, classification tasks is very high due to a lack of consensus in the field with respect to standardizing tasks, contrasts, and mental concepts [38]. In our experiments, we used the labels provided by NeuroVault as specified in the original studies [46, 48]. We chose to compare multiple types of classification on the HCP dataset to illustrate different approaches are in use in the field or that were used by other studies [28, 29]. For BrainPedia, a multilabel decoding was performed in the original study since multiple concepts are associated with most maps. Labels we had access to were then the list of labels associated with each map. To be able to compare our results with those of the homogeneous dataset (HCP), we chose to classify these as unique labels, which was less complex and less precise in practice. This type of issue is due to the lack of harmonization in the way tasks and cognitive processes are defined. Using ontologies such as Cognitive Atlas [38], NeuroVault annotations could be harmonized and enriched, as it was done by [37] by mapping the original labels to target ones from Cognitive Atlas or [61] in which cognitive conditions were annotated by a group of expert using the same atlas.

In neuroimaging, many sources of variability can impact the results of an experiment and the generalizability of the results. Here, we investigated the generalizability of our model by assessing the benefits of pretraining on a heterogeneous dataset (BrainPedia). While this dataset was heterogeneous in terms of the studies that were included, all maps were obtained using the same processing pipeline. Multiple studies have shown that the exact pipeline used to obtain an fMRI result can have a nonnegligible impact on fMRI statistic maps [62, 63]. In the future, investigating performance of classification on a more variable target dataset with statistic maps from different studies but also processed using different pipelines would be of great interest. In a recent study [10], the authors tried to compare the performance of different classifiers trained on fMRI 3D volumes series obtained with various scenarios of minimal preprocessing pipelines. A similar experiment was recently made by [64], who found that preprocessing pipeline selection can impact the performance of a supervised classifier. Comparing the adaptation capacities of models on volumes preprocessed with different pipelines could be also interesting to evaluate the impact of analytical variability on deep learning with fMRI and to see if the generalizability of our pretrained models also works for interpipeline differences.

Note that self-supervised (instead of self-taught) learning could have also been used to pretrain our model, as it was done by [33], who designed self-supervised learning frameworks, inspired by the field of natural language processing, to pretrain mental state decoding models. Self-supervised learning is a supervised machine learning setting where the supervision is generated directly from the data and the model is pretrained using a supervised surrogate task. Self-supervised learning is particularly relevant if the surrogate task is close to the final one targeted by the user (e.g., if they can share the same feature representation). It is possible that, by designing a relevant supervised surrogate task that could be relevant for all very diverse usage of our model, the pretrained model would have performed better than the one presented in this article. Designing and experimenting with such a surrogate supervised task could be interesting for future work.

In our self-taught context, using unsupervised models could allow us to build a space capturing the similarities and differences of statistic maps (i.e., to learn a robust latent representation of the important features of statistic maps in a specific context). By adding other constraints to this latent space and/or choosing an adapted pretraining dataset, we could use this for other purposes than brain decoding. For instance, building a space that captures the analytical variability in statistic maps could help us understand the difference between the pipelines but also identify the more robust pipelines. Future works will focus on building such a space with specific constraints to evaluate distance between different pipelines.

## Conclusion

In this study, we compared the benefits of a self-taught learning framework in the task of classifying 3D fMRI statistic maps. We showed that unsupervised pretraining improves the performance of classification in experiments with few training samples and complex classification tasks, which is a very common setup in fMRI studies.

## Data Availability

Supporting data, including supplementary materials (figures and tables) and additional links (code and derived data) is also available via the *GigaScience* repository, GigaDB [52]. The data used in this study are openly available on NeuroVault [36]. No experimental activity involving the human participants was made by the authors. Only publicly released data were used.

NeuroVault IDs of statistic maps included in each dataset (NeuroVault, HCP, and BrainPedia) are available in the Derived Data section.

## Supplementary Material

giad029_GIGA-D-22-00277_Original_Submission

giad029_GIGA-D-22-00277_Revision_1

giad029_GIGA-D-22-00277_Revision_2

giad029_GIGA-D-22-00277_Revision_3

giad029_Response_to_Reviewer_Comments_Original_Submission

giad029_Response_to_Reviewer_Comments_Revision_1

giad029_Response_to_Reviewer_Comments_Revision_2

giad029_Reviewer_1_Report_Original_Submission Huiguang He -- 11/2/2022 Reviewed

giad029_Reviewer_1_Report_Revision_1 Huiguang He -- 2/2/2023 Reviewed

giad029_Reviewer_2_Report_Original_Submission Yongjie Zhu -- 11/13/2022 Reviewed

giad029_Supplemental_Files

## Additional Files

**Supplementary Table S1 - validation_model_hcp.csv** - Classification performance (Accuracy and F1-score) of models trained on HCP validation dataset in a 5-fold cross-validation scheme with different hyperparameters (batch size, epochs, architecture).

**Supplementary Table S2 - validation_model_bp.csv** - Classification performance (Accuracy and F1-score) of models trained on BrainPedia validation dataset in a 5-fold cross-validation scheme with different hyperparameters (batch size, epochs, architecture).

**Supplementary Table S3 - test_hcp_contrast_sample_size.csv** - Classification performance (Accuracy and F1-score) of models trained on HCP test dataset with different subsamples in a 5-fold cross-validation scheme for contrast classification.

**Supplementary Table S4 - test_hcp_task_sample_size.csv** - Classification performance (Accuracy and F1-score) of models trained on HCP test dataset with different subsamples in a 5-fold cross-validation scheme for task classification.

**Supplementary Table S5 - test_hcp_one_contrast_task _sample_size.csv** - Classification performance (Accuracy and F1-score) of models trained on HCP test dataset with different subsamples in a 5-fold cross-validation scheme for one-contrast task classification.

**Supplementary Table S6 - comparison_bp.csv** - Classification performance (Accuracy and F1-score) of models trained on BrainPedia test dataset in a 5-fold cross-validation scheme.

**Supplementary Table S7 - comparison_small_bp.csv** - Classification performance (Accuracy and F1-score) of models trained on Small BrainPedia test dataset in a 5-fold cross-validation scheme.

**Supplementary Table S8 - per_class_accuracies.csv** - Classification performance per class of model trained on HCP dataset sample n=50 for the first fold.

**Supplementary Figure S1** - Schematic visualisation of the architectures of the Convolutional AutoEncoder (a) and Convolutional Neural Network (b) with 4 layers.

**Supplementary Figure S2** - Original mean statistic maps (column 1) and mean feature maps across subjects of the fold 1 of the test dataset of HCP 50 for the first four convolutional layers of each model (columns 2-4): CNN with default algorithm initialization (DA), pre-trained CNN (PT) and CAE for the 8 selected contrasts: `Working Memory’: `0-back body’, `0-back places’, `0-back tools’, `2-back body’, `2-back tools’ , `Gambling: punish', `Gambling: reward and `Relational.

## Abbreviations

2D: 2-dimensional; 3D: 3-dimensional; CAE: convolutional autoencoder; CNN: convolutional neural network; DL: deep learning; fMRI: functional magnetic resonance imaging HCP: Human Connectome Project; MSE: mean squared error; ReLU: rectified linear unit.

## Code

The code produced to run the experiments and to create the figures and tables of this article is available in the Software Heritage public archive at [43].

- Programming language: Python3.9
- Licence: MIT
- Requirements: numerous Python libraries described in the GitLab repository.

## Derived Data

Derived data such as NeuroVault IDs of statistic maps used in the different datasets and the parameters of the trained models (for validation of the hyperparameter and test of performance) are available in Zenodo: [44].
