# Supplementary material for: On the benefits of self-taught learning for brain decoding
Source: Gigascience. 2023 May 3;12:giad029. doi: 10.1093/gigascience/giad029 (PMC10155221; doi:10.1093/gigascience/giad029)
Supplement: giad029_GIGA-D-22-00277_Original_Submission [file giad029_giga-d-22-00277_original_submission.pdf]

GigaScience

On the benefits of self-taught learning for brain decoding

--Manuscript Draft--

**Manuscript Number:**	GIGA-D-22-00277
**Full Title:**	On the benefits of self-taught learning for brain decoding
**Article Type:**	Research
**Funding Information:**	Région Bretagne (ARED MAPPIS) — Ms. Elodie GERMANI<br>Agence Nationale de la Recherche (ANR-20-THIA-0018) — Ms. Elodie GERMANI
**Abstract:**	We study the benefits of using a large public neuroimaging database composed of fMRI statistic maps, in a self-taught learning framework, for improving brain decoding on new tasks. First, we leverage the NeuroVault database to train, on a selection of relevant statistic maps, a convolutional autoencoder to reconstruct these maps. Then, we use this trained encoder to initialize a supervised convolutional neural network to classify tasks or cognitive processes of unseen statistic maps from large collections of the NeuroVault database. We show that such a self-taught learning process always improves the performance of the classifiers but the magnitude of the benefits strongly depends on the number of samples available both for pre-training and finetuning the models and on the complexity of the targeted downstream task.
**Corresponding Author:**	Elodie GERMANI<br>Universite de Rennes 1<br>Rennes, FRANCE
**Corresponding Author Secondary Information:**	
**Corresponding Author's Institution:**	Universite de Rennes 1
**Corresponding Author's Secondary Institution:**	
**First Author:**	Elodie GERMANI
**First Author Secondary Information:**	
**Order of Authors:**	Elodie GERMANI<br>Elisa Fromont, Professor<br>Camille Maumet, PhD
**Order of Authors Secondary Information:**	
**Additional Information:**	
**Question**	**Response**
Are you submitting this manuscript to a special series or article collection?	No
**Experimental design and statistics**<br><br>Full details of the experimental design and statistical methods used should be given in the Methods section, as detailed in our Minimum Standards Reporting Checklist. Information essential to interpreting the data presented should be made available in the figure legends.	Yes

Powered by Editorial Manager® and ProduXion Manager® from Aries Systems Corporation

Have you included all the information requested in your manuscript?	
**Resources**<br><br>A description of all resources used, including antibodies, cell lines, animals and software tools, with enough information to allow them to be uniquely identified, should be included in the Methods section. Authors are strongly encouraged to cite Research Resource Identifiers (RRIDs) for antibodies, model organisms and tools, where possible.<br><br>Have you included the information requested as detailed in our Minimum Standards Reporting Checklist?	Yes
**Availability of data and materials**<br><br>All datasets and code on which the conclusions of the paper rely must be either included in your submission or deposited in publicly available repositories (where available and ethically appropriate), referencing such data using a unique identifier in the references and in the "Availability of Data and Materials" section of your manuscript.<br><br>Have you have met the above requirement as detailed in our Minimum Standards Reporting Checklist?	Yes

Powered by Editorial Manager® and ProduXion Manager® from Aries Systems Corporation

Manuscript

Click here to access/download;Manuscript;on_the_benefits_of_self_taught_lea

On the benefits of self-taught learning for brain decoding

Elodie Germani
Univ Rennes, Inria, CNRS, Inserm
Rennes, France
elodie.germani@irisa.fr

Elisa Fromont *
Univ Rennes, IUF, Inria, CNRS, IRISA
Rennes, France

Camille Maumet *
Univ Rennes, Inria, CNRS, Inserm
Rennes, France

Abstract

We study the benefits of using a large public neuroimaging database composed of fMRI statistic maps, in a self-taught learning framework, for improving brain decoding on new tasks. First, we leverage the NeuroVault database to train, on a selection of relevant statistic maps, a convolutional autoencoder to reconstruct these maps. Then, we use this trained encoder to initialize a supervised convolutional neural network to classify tasks or cognitive processes of unseen statistic maps from large collections of the NeuroVault database. We show that such a self-taught learning process always improves the performance of the classifiers but the magnitude of the benefits strongly depends on the number of samples available both for pre-training and finetuning the models and on the complexity of the targeted downstream task.

Keywords Self-taught Learning · Brain Decoding · Autoencoder · Convolutional Neural Network · Deep Learning

1 Introduction

In the past few years, deep learning (DL) approaches have achieved outstanding performance in the field of neuroimaging [1] due to their ability to model complex non-linear relationships in the data. Functional Magnetic Resonance Imaging (fMRI) data, a noninvasive neuroimaging technique in which brain activity is recorded during specific experimental protocols probing different mental processes and giving a big picture on cognition, are often used as input data to these models. These can be used for different purpose, such as disease diagnosis [2] or brain decoding (*i.e.* identifying stimuli and cognitive states from brain activities) [3], with a common goal: linking a target with highly variable patterns in the data and ignoring aspects of the data that are unrelated to the learning task. Researchers took advantage of the specific properties of fMRI data to build more and more sophisticated models [4, 5, 6, 7, 8, 9, 10, 11].

However, training effective DL models using fMRI data comes with many challenges [12]. Among these is the need for large amounts of training data to compensate for the large number of trainable parameters in DL models [13]. Performance of these models are limited by the high dimensionality and low sample size of conventional fMRI datasets [14, 15]. These are typically composed of 3D volumes with hundred thousand dimensions (or voxels) for each for a rather small number of subjects (typically 10-100). Although sample sizes in fMRI studies increased these past few years and despite the growing efforts in collecting large-scale fMRI datasets [16], these studies often focus on a small number of specialised tasks. Thus, each neuroimaging experiment only explore a few cognitive processes, making it difficult to build models that can effectively be applied to new studies.

To prevent overfitting and allow for generalizable statistical inference, researchers focused on methods to tackle this lack of training data [17, 18, 19]. For instance, [20] built a decoding model using data gathered from 35 studies and

*Joint senior authorship

thousands of individuals that cover various cognitive domains. Despite the good performance of the models, these can only be applied on restricted sets of studies, discriminating between few cognitive concepts. More annotated training data (*e.g.* using large public databases) would be required to map a wider set of cognitive processes.

This problem of small training sets is not limited to neuroimaging and is well known in the field of machine learning, where researchers extensively use deep transfer learning to improve classification and generalization performance of their models [21]. It consists in using the knowledge obtained from a model trained for a source task on a source dataset and applying it to a target task on a target dataset. Transfer learning proved its worth by using large, publicly available datasets [22] to pre-train DL models before fine-tuning them on smaller datasets of a related domain.

In neuroimaging, lots of studies were made on inductive transfer learning with labeled source data as defined in [21] (e.g. source task and target task are different, as well as source domain and target domain) [23, 24, 25]. For instance, [23] pre-trained two DL classifiers on a large, public whole-brain fMRI dataset of the HCP, fine-tuned them and evaluated their performance on another task on the same dataset and on a fully independent dataset. In another study, [24] used the ImageNet database [22], a large, public dataset containing naturalistic images from more than 1000 classes, to pre-train a model and adapt it to classify tasks from 2D fMRI data. This database was also used in [26] for pre-training a 2D structural MRI classifier. In the same paper, the Kinetics dataset [27] was also used to evaluate the transfer learning process with 3D images. In a recent work, [28] used self-supervised learning frameworks to pre-train DL decoding models across a broad fMRI dataset, comprising many individuals, experimental domains, and acquisition sites. These studies showed improved classification accuracies as well as quicker learning and less training data required.

However, labeled databases are not always available in neuroimaging. Despite the growing effort in data sharing to build public databases [29], such as OpenNeuro for raw data [30] and NeuroVault for fMRI statistic maps [31]. The unconstrained annotations and the heterogeneity of tasks and studies make them difficult to use to pre-train a supervised deep learning model. To compensate this, weakly supervised learning techniques such as automatic labelling of data has proved its worth. For instance, [32] enriched NeuroVault annotations using the Cognitive Atlas ontology [33] and used these labeled data to train a multi-task decoding model that successfully decoded more than 50 classes of mental processes on a large test set.

A specific type of inductive transfer learning named *self-taught learning* [34, 35] showed strong empirical success in the field of machine learning. It does not require any labels as it consists in training models to autonomously learn latent representations of the data and using these to improve learning in a supervised setting. This approach is motivated by the observation that data from similar domains contain patterns that are similar to those of the target domain. By initializing the weights of a supervised classifier with the pre-trained weights of an unsupervised model trained on many images. The aim is to improve the model performance by placing the parameters close to a local minimum of the loss function and by acting as a regularizer [36].

In the field of neuroimaging, latent representations have recently been used in a task-relevant autoencoding framework. [37] used an autoencoder with a classifier attached to the bottleneck layer on a small fMRI dataset. This model outperformed the classifier trained on raw input data by focusing on cleaner, task-relevant representations. This suggests that a low-level representation of fMRI data, learned for a reconstruction task, could be helpful in a classification task, as in a self-taught learning framework.

In this work, we want to take advantage of a large public neuroimaging database in a self-taught learning framework. We pre-train an unsupervised deep learning model to learn a latent representation of fMRI statistic maps and we fine-tune this model to decode tasks or mental processes involved in several studies. In a first part, we leverage the NeuroVault database to select the most relevant statistic maps and train a convolutional autoencoder (CAE) to reconstruct these maps. In a second part, we use the final weights of the encoder to initialize a supervised convolutional neural network (CNN) to classify the cognitive processes, tasks or constrasts of unseen statistic maps from large collections of the NeuroVault database (an homogeneous collection of more than 18000 statistic maps and an heterogeneous one with 6500 maps). Our goal was to investigate how self-taught learning can be beneficial in the field of brain imaging for deep learning models.

2 Material & Methods

The code produced to run the experiments and to create the figures and tables of this paper is available in the Software Heritage public archive `https://archive.softwareheritage.org/browse/origin/https://gitlab.inria.fr/egermani/self_taught_decoding.`. Derived data used by these notebooks are stored in Zenodo [38].

Figure 1 illustrates the overall process used to implement our self-taught learning framework: a CAE is first trained to reconstruct the maps of a large dataset extracted from NeuroVault. Then, the encoder part of the CAE is fine-tuned to answer a classification problem on another dataset (with labels). After hyperparameters optimisation, performance of

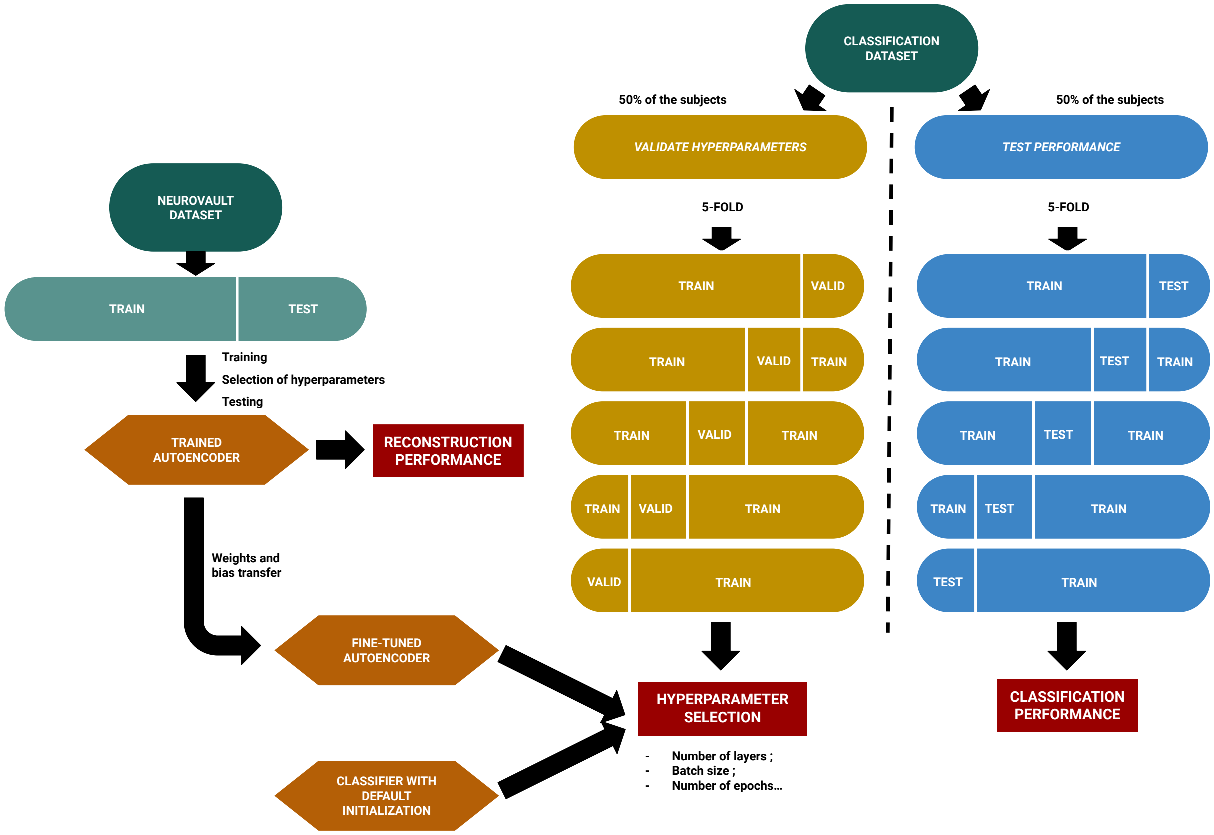

Figure 1: Flow diagram of the self-taught learning methodology. NeuroVault dataset was used to train a Convolutional AutoEncoder (CAE). The encoder of this CAE was used to initialize a Convolutional Neural Network (CNN) and train it to classify other datasets. These classification datasets are split in two disjoints datasets: a ‘validation’ one used to optimize hyperparameters and a ‘test’ one to evaluate performance. In each one, a 5-fold cross-validation was performed.

the pre-trained classifier are compared to those of a classifier initialized with a default algorithm. Details regarding the datasets (NeuroVault dataset and classification datasets) can be found in the next subsection. The models of the CAEs and the CNNs are presented in Section 2.3. Further explanations on the workflow used to train the CAE and the CNN and to evaluate their performance are available in Sections 2.4 and 2.5 respectively.

2.1 Overview of the datasets

A summary of the different datasets can be found in Table 1. Details are given below.

Table 1: Overview of the datasets. For each dataset, number of statistic maps are presented, as well as the number of subjects, number of studies and the type of labels (if available).

Dataset	Maps	Subjects	Studies	Labels
NeuroVault	28,532	-	-	-
HCP	18,070	787	1	Tasks (*7*)<br>Contrasts (*23*)
BrainPedia	6,448	826	29	Cognitive processes (*36*)

2.1.1 NeuroVault dataset

NeuroVault [31] (RRID:SCR_003806) is a web-based repository for statistic maps, parcellations and atlases produced by MRI and PET studies. This is currently the largest public database of fMRI statistic maps. NeuroVault has its own public Application Programming Interface (API) that provides a full access to all images (grouped by collections) and enables filtering of images or collections with associated metadata. At the time of experiment (19/01/2022), a total of 461,461 images in 6,782 collections were available. Among the available metadata, some were mandatory and specified for all maps such as the modality (e.g. "fMRI-BOLD" for Blood-Oxygen Level Dependent Functional MRI; dMRI for Diffusion MRI, etc.), the type of statistic (e.g. "T map" or "Z map") or the cognitive paradigm (e.g. "Working memory" or "Motor fMRI task paradigm"), and others were optional and only available if additionally entered at the time of the upload.

From this large database, relevant maps were selected based on multiple criteria. First, we chose maps for which the modality was 'fMRI-BOLD' to exclude others modalities such as structural or diffusion MRI. To get comparable maps, we set three additional inclusion criteria and selected maps: 1/ for which all required metadata were provided ('is_valid' to True) 2/ that were registered in MNI space ('not_mni' to False) – to ensure that anatomical structures were located at the same coordinates in each map – and 3/ referenced as 'T map' or 'Z map' – to exclude maps in which voxel values did not have the same meaning (e.g. P value maps, Chi-squared maps, etc.) –. Among these, thresholded statistic maps were excluded.

We found that some maps in our initial dataset, were wrongly referenced as T map or Z map. These misclassified maps were removed by filtering the 'filename' column of the dataframe to exclude *SetA_mean SetB_mean* (AFNI contrast maps), *con* (SPM contrast maps), *cope* (FSL contrast maps).

Using these criteria, a total of 28,532 statistic maps were selected from the NeuroVault database and constituted our 'NeuroVault dataset'. Most of these maps were unlabeled (*i.e.* cognitive processes or tasks performed described as 'None / Other') or not labeled in a standardized way (*i.e.* use of terms that are specific for a study instead of generic terms, such as those defined in [33] *e.g.* some maps are labeled as 'word-picture matching task' for the cognitive paradigm whereas others in which a similar task is performed are referenced as 'working memory fMRI task paradigm' which is a label that includes other specific tasks).

2.1.2 HCP dataset (NeuroVault Collection 4337)

NeuroVault collection 4337 [39] included 18,070 z-statistic maps, for base contrasts (task vs baseline), corresponding to 787 subjects of the HCP 900 release [16]. This collection was excluded from our pre-training dataset (see section 2.1.1) due to missing metadata (i.e. 'is_valid' was False).

All maps in this collection were grouped together and referred to as the 'HCP dataset' in the following. Multiple labels were entered for each map including: mental concepts ('cognitive_paradigm_cogatlas'), tasks ('task') and contrasts ('contrast_definition') (as defined in [33]). For each subject, 23 contrasts ('0BKBODY', '0BK-FACE', '0BKPLACE', '0BKTOOL', '2BKBODY', '2BK-FACE', '2BKPLACE', '2BKTOOL', 'CUE', 'FACES', 'LF', 'LH', 'MATCH', 'MATH', 'PUNISH', 'RANDOM', 'REL', 'REWARD', 'RF', 'RH', 'SHAPES', 'STORY', 'TOM') distributed in 7 tasks ('EMOTION', 'GAMBLING', 'LANGUAGE', 'MOTOR', 'RELATIONAL', 'SOCIAL', 'WM') were available. For more details on contrasts, tasks and mental concepts of this study, see [16].

2.1.3 BrainPedia dataset (NeuroVault collection 1952)

NeuroVault collection 1952 [40], known as BrainPedia [41], contained fMRI statistic maps of about 30 fMRI studies from OpenNeuro [30], the Human Connectome Project [16] and from data acquired at Neurospin research center, together they were chosen to map a wide set of cognitive functions.

This collection contained 6,573 statistic maps corresponding to 45 unique mental concepts derived from 19 sub-terms (*e.g.* 'visual, right hand, faces' for maps associated with the task of watching an image of a face and responding to a working memory task). These images were previously used to build a multi-class decoding model [41] and labels corresponded to the mental concepts associated with the statistic map, e.g., 'visual', 'language' or 'objects'. Here we excluded the nine classes that had less than 30 samples each, leaving 6,448 images corresponding to 36 classes. These 6,448 images were grouped together and referred to as the 'BrainPedia' dataset in the following.

2.2 Preprocessing

All statistic maps included in this study were downloaded from different collections of NeuroVault and therefore were obtained using different processing pipelines (see the original studies for more details [41], [16]). In addition,

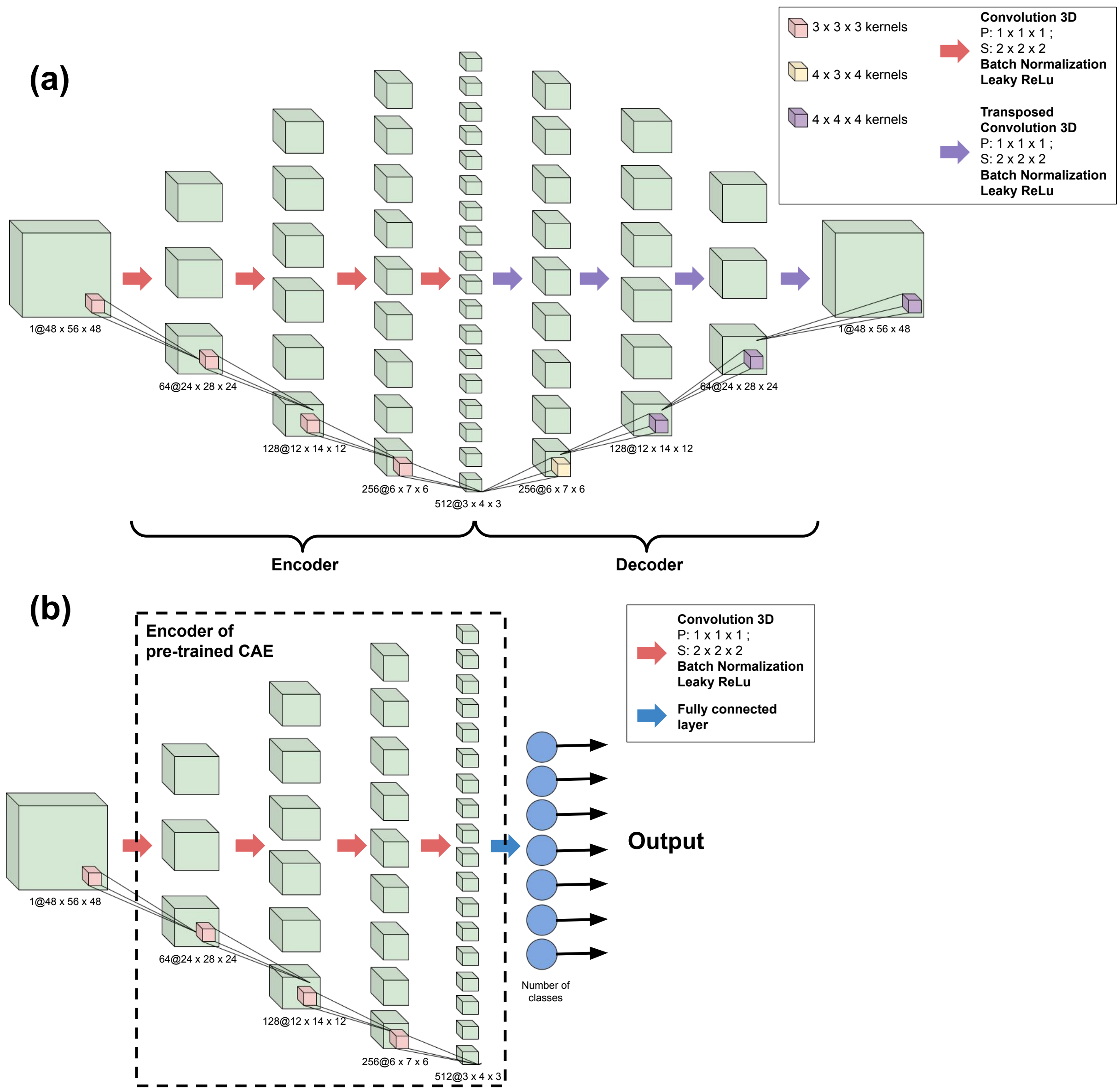

Figure 2: Schematic visualisation of the architectures of the Convolutional AutoEncoder (a) and Convolutional Neural Network (b) with 4 layers. The CAE is composed of an encoder and a decoder with respectively 4 convolutional and transposed convolutional layers. The size of the latent space is 512 * 3 * 4 * 3. The CNN has the same architecture as the encoder of the CAE with a fully-connected layer added at the end of the network with different numbers of output node depending on the dataset and the classification performed.

we resampled all maps to dimensions (48, 56, 48) using the MNI152 template available in Nilearn [42] (RRID: SCR_001362) as target image. A min-max normalization was also performed on all resampled maps to get statistical values between -1 and 1. Finally, the brain mask of the MNI152 template in Nilearn was used to exclude statistical values outside the brain in all statistic maps.

2.3 Model architectures

All models were implemented using PyTorch [43] v1.12.0 (RRID:SCR_018536) with CUDA [44] v10.2. For our model architectures, we chose to use 3D-convolutional feature extractors that take into account the three spatial dimensions of

fMRI statistic maps. Schematic representations of the architectures are available in Figure 2 and Supplementary Figure S1.

2.3.1 Convolutional AutoEncoder (CAE)

The base architecture of our CAE was inspired from [19]. Two architectures were derived from this base: a 4 layers and a 5 layers architecture, respectively corresponding to the number of convolutional layers in each part of the CAE (encoder and decoder). In the 4-layer model, the encoder part consisted in four 3D convolutional layers with respectively 64, 128, 256 and 512 channels. Each layer had a kernel size of 3 x 3 x 3, a stride of 2 x 2 x 2 and a padding of 1 x 1 x 1. 3D batch normalization layers [45] followed each convolutional layers with respectively 64, 128, 256 and 512 channels and a leaky rectified linear unit (ReLU) activation function was used for all layers. The decoding part of the CAE was symmetric to the encoder, except that 3D transposed convolutional layers were used instead of classic convolutional layers. Transposing convolutions is a method to upsample an output using learnable parameters. It can be seen as an opposite process to classical convolutions. To keep the number of features symmetric at each layers output, the kernel size of the first layer was set to 4 x 3 x 4 and to 4 x 4 x 4 for all other transposed convolutional layers. Leaky ReLU activation function was also used for all layers except for the last one, *i.e.* the output one, for which a sigmoid function was used in order to obtain output values between -1 and 1. The latent space for this model was of size 512 x 3 x 4 x 3. A schematic representation of this architecture can be found in Figure 2(a).

In the 5-layer model, one convolutional layer was added at the beginning of the encoder with 32 channels and similar parameters as the other layers of the encoder. A transposed convolutional layer was also added at the end of the decoder with 32 channels. The kernel sizes in the decoder were also modified to maintain the feature map sizes: the first and second layers of the decoder had kernel sizes of 3 x 4 x 3 and 4 x 3 x 4 respectively. All other parameters, batch normalization layers and activation functions were the same. The latent space for this model was of size 512 x 2 x 2 x 2. A schematic representation of this architecture can be found in the Supplementary Figure S1 (a).

2.3.2 Convolutional Neural Network (CNN)

The 3D CNNs used for classification followed the architecture of the encoder part of the CAEs. In the same way as for the CAEs, two CNN architectures were derived. For each one, we took the corresponding architecture of the encoder (4 or 5 layers) and added a fully connected layer at the end. The number of nodes in this layer varied depending on the number of classes. A softmax activation function was used for this output layer. Visual representation of the CNNs are available in Figure 2(b) and Supplementary Figures S1 (b).

2.4 CAE training

To train our CAEs to reconstruct the statistic maps of the NeuroVault dataset, we used an Adam optimizer [46] with a learning rate of 1e-04 and all other parameters with default values. The loss function was the Mean Squared Error (MSE: the squared L2 norm) which is the standard reconstruction loss.

2.4.1 Dataset split

NeuroVault dataset was randomly split in two subsets: training and test with respectively 80% and 20% of the maps. The training set (N=22,772 maps) was used to train the CAE with the different architectures and the test set (N=5,760 maps) to assess the performance of the different models (with different hyperparameters).

2.4.2 Architecture comparison

To limit the computational cost of our experiments, we fixed some of the hyperparameters of the CAE and only compared those who were of interest for the later experiments. Here, we use the term model "hyperparameters", to distinguish with model "parameters", to represent the values that cannot be learnt during training, but are set beforehand e.g., the batch size or the number of hidden layers. Thus, a batch size of 32 and a learning rate of 1e-04 were chosen to train the CAE for a number of 200 epochs (*i.e.* values that are often used in experiments). The only hyperparameter for which different values were compared was the number of hidden layers of the model: 4 layers vs 5 layers for each part (encoder/decoder) of the model.

2.4.3 Performance evaluation

To assess the performance of the CAEs, we estimated Pearson's correlation coefficient between the reconstructed statistic map and the original statistic map. The correlation coefficient was computed using numpy version 1.21.2 (RRID: SCR_008633)[47]. The closer to 1 the correlation coefficient was, the stronger the relationship between the

maps and the more accurate the reconstruction. Note that we did not use MSE in this context as its individual values (for each data point) are not easily interpreted.

2.5 Classifier training

We trained two types of classifiers for all the experiments:

- the *classifier with default algorithm* initialized with the original algorithm from [48] (*i.e.* Kaiming Uniform algorithm for convolutional and fully-connected layers with a parameter of $\sqrt{5}$) and
- the *classifier with pre-trained CAE* initialized using the weights and bias of the convolutional layers of the CAE pre-trained on NeuroVault dataset.

The CNNs were trained using the Adam optimizer with a learning rate of 1e-04. We used the cross-entropy loss function for training the classifier. Both were implemented in PyTorch.

2.5.1 Dataset split

As described in Fig. 1 (on the right), the classification datasets were split in two disjoint subsets: the *'validation dataset'* used to optimize the hyperparameters, and the *'test dataset'* used to test the performance. Each subset contains 50% of the subjects of the overall dataset with no overlap to avoid any data leakage (see [49, 50]).

For each experiment, the validation and test datasets were then split into 5 folds for cross-validation. Subjects were randomly sampled in each fold in order to ensure that there was no overlap of subjects across folds. The identifiers of the subjects included in the different folds were saved for reproducibility. More details on the methods used to perform the 5-folds split for each dataset are specified in subsection 2.6.

2.5.2 Evaluation of performance

The performance of each model was measured using several metrics: accuracy (Acc), precision (P), recall (R) and F1-macro score (F1). All metrics were implemented using scikit-learn [42] with default parameters, except for F1-score for which the "average" parameter was specified with "macro" to deal with multi-class classification.

To evaluate the performance of a model, all metrics were averaged among the 5 folds of cross-validation and standard error of the mean was computed.

To compare the final performance of models with default initialization versus fine-tuned weights, we used paired two-sample t-tests between the performance values (accuracy or F1-score) of the 5 models trained during cross-validation. T-statistic and p-value were provided and value of 0.05 was used for the p-value significance threshold.

2.5.3 Hyperparameters optimisation

To select the best hyperparameters for each dataset and each type of initialization, we evaluated the performance of each model by performing a 5-fold cross-validation on the validation dataset.

For each type of classifier (i.e. initialized with default algorithm versus pre-trained), we refined and optimised the hyperparameters using the largest datasets (Large BrainPedia and HCP). However, the large amount of training data makes it computationally extremely costly to perform a full grid-search. We therefore limited our research to predefined values of batch sizes (32 or 64), number of epochs (200 or 500) and model architectures (4 layers or 5 layers). All batch sizes, number of epochs and architectures were tested for each type of classifier and each dataset. We didn't perform any optimization on the learning rate to limit the computational cost of our experiments. Every model was trained using a learning rate of 1e-04.

We selected the best set of hyperparameters based on the performance of the corresponding model in terms of accuracy and F1-score, averaged across folds.

2.6 Benefits of self-taught learning and impact of different factors

To investigate the benefits of self-taught learning for neuroimaging data, different brain decoding experiments were studied. For all, after optimizing the hyperparameters of the two models (i.e. the model with default initialization -or- with pre-trained CAE and fine-tuned weights) we assessed the performance of these optimized models on the test dataset using a 5-fold cross-validation.

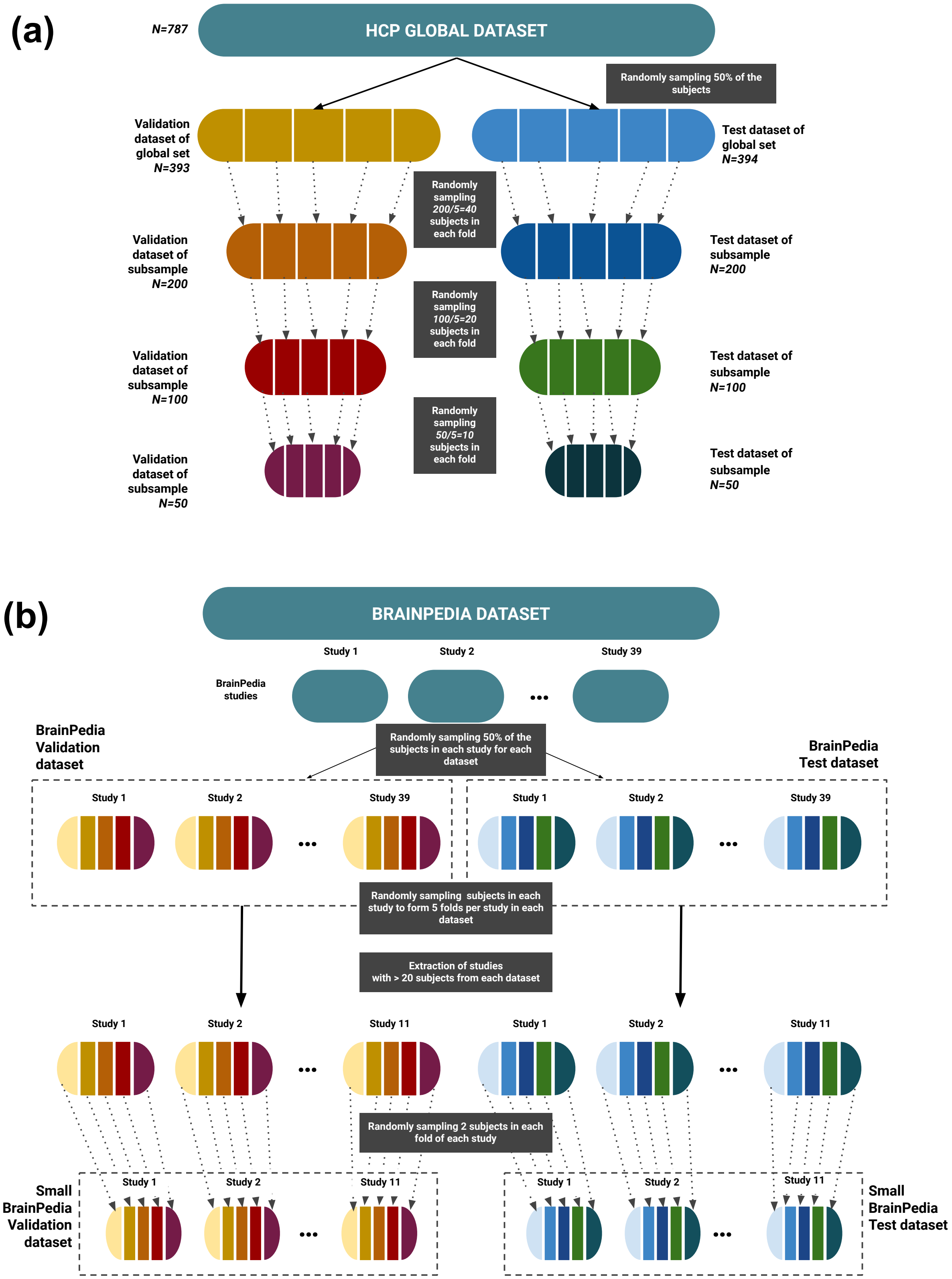

Figure 3: Overview of the process used to split the datasets for cross-validation. (a) shows the method performed for HCP dataset and its subsamples and the one used for BrainPedia and Small BrainPedia datasets is presented in part (b). In both cases, the global dataset was first split into two subdatasets ‘validation’ and ‘test’ with respectively 50% of the subjects and then each subdataset was divided into 5 folds for cross-validation.

2.6.1 Homogeneous dataset (single study)

The HCP dataset was used to compare the performance of the models for the task of decoding on a homogeneous dataset (*i.e.* from a single study). We studied the impact of two factors on the classification: sample size and number of target classes. For sample size, subsets of the global HCP dataset were created with different number of subjects: N=50, 100 and 200. Each smaller subset being a subset of the immediately larger one. To create these subsets, we first split the global HCP test dataset into 5 folds, with different subjects in each fold. In each of these 5 folds, we randomly sampled $200/5 = 40$ subjects and obtained 5 sub-folds that together composed the smaller subset of 200 subjects. This process was repeated for subsamples N=100 and 50 by sampling from their superset. This insured that the 5 models trained on different combinations of the 4 folds of a smaller subset could be tested on the remaining fold of the global test dataset with no overlap between the training and test data. The process is illustrated in Fig. 3(a).

Three types of classification were investigated. First, the 'contrast classification' which consisted in identifying the contrast associated with a statistic map (23 different contrasts). Second, the 'task classification' which consisted in identifying the task associated with a statistic map (7 different tasks, with multiple contrasts per task). Third, the 'one contrast task classification'. This time, we selected a single contrast per task and classified the tasks (7 different tasks, with one contrast per task). The selected contrasts were '2BKPLACE', 'FACES', 'PUNISH', 'REL', 'RH', 'STORY' and 'TOM' respectively for the tasks 'WM', 'EMOTION', 'GAMBLING', 'RELATIONAL', 'MOTOR', 'LANGUAGE', 'SOCIAL'. The dataset used for this third type of classification was thus smaller than the others (only one map per task per subject).

2.6.2 Heterogeneous dataset (multiple studies)

To study the benefits of self-taught learning on a heterogeneous dataset (i.e. from multiple studies), we used BrainPedia. For these experiments, we focused on the classification of mental concepts (as available in NeuroVault metadata). Fig. 3(b) illustrates the process used to split this dataset. To perform the split while maintaining the heterogeneity in each fold, we randomly sampled 50% of the subjects of each study to form the 'validation' and 'test' datasets of BrainPedia (see Fig 3 b.). Then, each dataset, each study was split into 5-folds and the n-th folds of the different studies were combined to form the n-th fold of the dataset.

We also studied the impact of sample size in the presence of heterogeneity by extracting smaller datasets. Among the 29 studies of the BrainPedia dataset, we only kept those which were composed of more than 20 subjects. In these remaining studies, already split into 5 folds in BrainPedia validation and test subdatasets, 2 subjects were randomly drawn per fold per study per subdataset to obtain 10 subjects per study per subdataset. Like above, the n-th folds of the different studies were combined to form the n-th fold of each subdataset of the 'Small BrainPedia' dataset. In the end, this smaller dataset was composed of 1,844 maps, divided in 30 classes, from 11 studies and 220 subjects.

3 Results

3.1 AutoEncoder performance

Reconstruction performance of the CAEs are presented in Table 2. When comparing the two CAE architectures (4-layers vs 5-layers) trained on NeuroVault dataset, the mean correlations between original and reconstructed maps are better for the 4-layers architecture (86.9% vs 77.8%). These results suggest that the reconstruction capabilities of the CAEs are dependant on the model architecture and the size of the latent space. Figure 4 shows the reconstruction of a statistic map randomly drawn from the NeuroVault test dataset with the two CAE architectures. With the 4-layers architecture, details of the map are better reconstructed than with the 5-layers architecture (see the green square on the map). This is due to the level of compression of the data that is higher in the 5-layers CAE and that learn only the most useful features with less emphasis in learning specific details. Both models were used as pre-trained model for classification to see if the benefits of the CAEs were related to their reconstruction performance.

Table 2: Reconstruction performance of the CAE depending on model architecture and training set. Values are the mean Pearson's correlation coefficients (standard error of the mean).

**Model**	**4-layers** *Latent space 18,432*	**5-layers** *Latent space 4,096*
Correlation (*std error*)	86.9 (*0.18*)	77.8 (*0.23*)

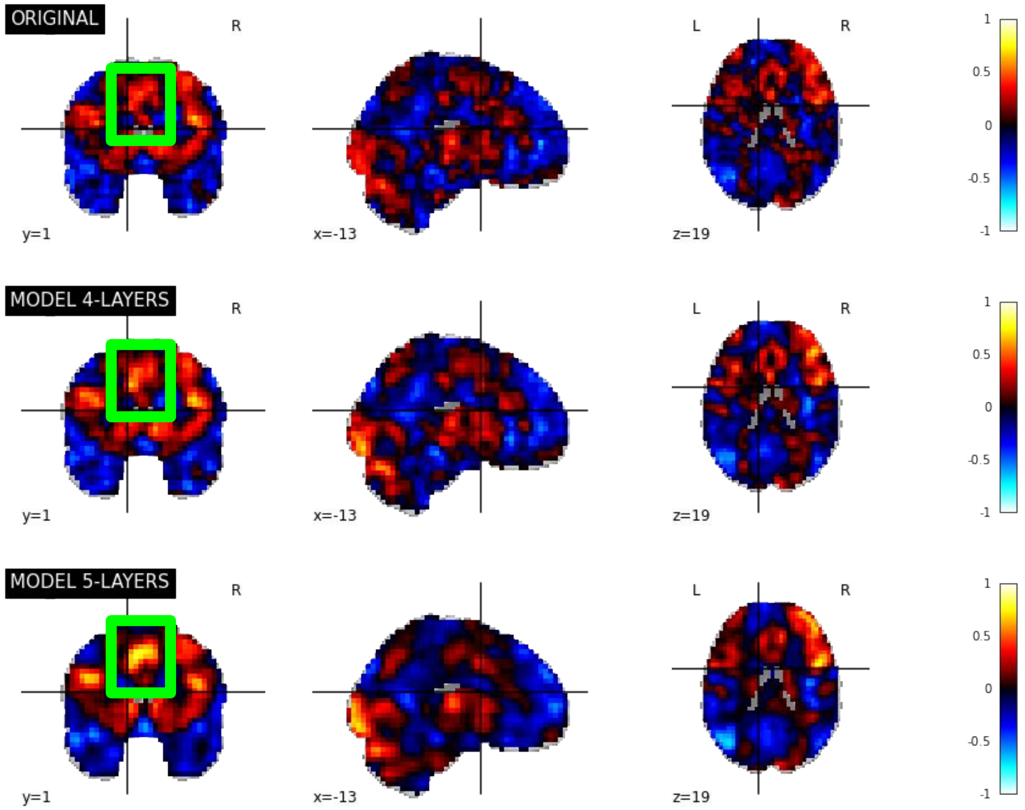

Figure 4: Original version and reconstruction of a randomly drawn statistic map of NeuroVault test dataset (image ID: 109) with the two CAEs (4-layers and 5-layers).

3.2 Hyperparameters optimisation for classifiers

The best hyperparameters and corresponding performance can be found on Table 3.

Table 3: Hyperparameters chosen for each dataset and corresponding performance of the classifier on the validation set of the dataset.

Dataset	Initialization	Model	Epochs	Batch	Average Accuracy (%) (*std. err.*)	Average F1 (%) (*std. err.*)
HCP	Default algorithm	4-layers	500	32	90.8 (*1.5*)	90.8 (*1.6*)
	Pre-trained CAE	5-layers	200	64	91.8 (*0.9*)	91.8 (*0.9*)
BrainPedia	Default algorithm	5-layers	500	64	67.1 (*1.7*)	61.0 (*1.6*)
	Pre-trained CAE	5-layers	200	64	73.8 (*2.7*)	70.0 (*2.3*)

3.2.1 Choice of hyperparameters for HCP dataset

Performance of the different models trained with the different hyperparameters can be found in Supplementary Table S1. For the default algorithm initialization, the best model had 4 layers and was trained with a batch size of 32 for 500 epochs. This model achieved an accuracy of 90.8% on average of the 5-folds of cross-validation. For the pre-trained

CAE initialization, the best model had 5 layers and was trained with a batch size of 64 for 200 epochs (average accuracy of 91.8%). The best hyperparameters for each type of initialization (default and pre-trained) were used in all subsequent experiments.

3.2.2 Choice of hyperparameters for BrainPedia dataset

Results for all sets of hyperparameters are available in Supplementary Table S2. For the default algorithm initialization, the model who achieved the best performance had 5 layers and a batch size of 64 for 500 epochs. This model classified the BrainPedia dataset with an average accuracy of 67.1% and an average F1-score of 61%. The performance of the pre-trained CAE was the best using a 5-layer architecture, a batch size of 64 and a training time of 200 epochs.

3.3 Benefits of self-taught learning on a homogeneous dataset

Table 4 summarizes the results for the different classification experiments on the HCP datasets.

Table 4: Classification performance on HCP datasets of models initialized with default algorithm vs with the weights of a pre-trained CAE. Mean accuracies and standard errors of the means among the 5-folds of cross-validation are shown. Paired two samples t-tests were performed between the accuracies of the 5 models obtained with cross-validation for each type of initialization. DA: Default Algorithm initialization ; PT: Pre-Training initialization.

**Sample**	**50**		**100**		**200**		**Global**	
**Init.**	**DA**	**PT**	**DA**	**PT**	**DA**	**PT**	**DA**	**PT**
**Contrast classification** (*23 classes*)								
Mean Acc. (%)	83.6	87.0	86.8	89.9	88.6	90.2	90.9	92.4
(std. err.)	(0.61)	(0.51)	(0.69)	(0.34)	(0.84)	(1.46)	(0.38)	(0.44)
Paired T-test (*4 dof*)		**-11.52**		**-4.77**		-1.42		**-4.74**
p-value		***0.0003***		***0.009***		*0.23*		***0.009***
**Task classification** (*7 classes, multiple contrasts per class*)								
Mean Acc. (%)	96.6	97.3	95.4	98.0	97.9	98.5	98.4	99.0
(std. err.)	(0.47)	(0.43)	(1.49)	(0.25)	(0.44)	(0.16)	(0.17)	(0.13)
Paired T-test (*4 dof*)		***-3.57***		-1.4		-1.5		***-5.65***
p-value		***0.02***		*0.2*		*0.2*		***0.005***
**One constrast task classification** (*7 classes, one contrast per class*)								
Mean Acc. (%)	97.9	99.1	98.9	99.4	99.3	99.6	99.4	99.6
(std. err.)	(0.3)	(0.3)	(0.17)	(0.25)	(0.2)	(0.2)	(0.2)	(0.14)
Paired T-test (*4 dof*)		**-4.17**		**-3.32**		-2.33		-2.06
p-value		***0.01***		***0.03***		*0.08*		*0.1*

3.3.1 Impact of sample size

For all classification experiments, the size of the training set (in terms of number of subjects) had a strong impact on the benefits of self-taught learning. With 50 subjects, the performance of the pre-trained CAE outperformed the performance of the classifier initialized with the default algorithm in all our experiments (improvements of 0.7% to 3.4% in mean accuracies). These improvements were always significant (P-values < 0.05). When sample size increased, this improvement reduced and was sometimes not significant. If we focus on contrast classification (Figure 5), which is the

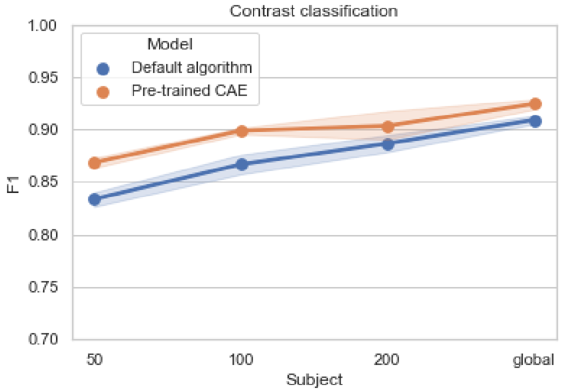

Figure 5: Mean accuracies and standard errors of the mean on contrast classification with the HCP dataset for the models initialized with default algorithm (blue) and pre-trained CAE (orange). Pre-training improves contrast classification performance for small sample sizes and at a lower level of improvement, also for large sample sizes.

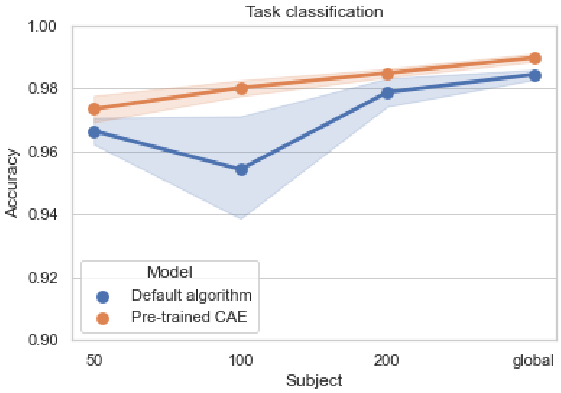

Figure 6: Mean accuracies and standard errors of the mean on task classification with the HCP dataset for the models initialized with default algorithm (blue) and pre-trained CAE (orange). Pre-training improves task classification performance for all sample sizes but sample sizes does not have a huge influence on the level of improvement.

hardest classification task between the three presented here due to the higher number of classes, the difference between the performance of the two classifiers decreased with sample size (mean accuracies of 88.6% and 90.2% respectively for default initialization and pre-trained model respectively for N=200 which corresponds to an improvement of 1.6% compared to almost 3% for N=100). For N=200, the difference of performance was not significant, probably due to the presence of an outlier value in the accuracies of the pre-trained CAE. Indeed, accuracies of the pre-trained CAE model are superior to the ones of the default model, except for the pre-trained model tested on the 3rd fold of cross-validation which was lower. This value was also significantly lower than those of models tested on other folds of cross-validation (see Supplementary Table S3).

3.3.2 Impact of the target classification task

For simpler classification experiments (*i.e.* with less classes to separate), pre-training was not always useful. In these experiments, performance were already nearly perfect (accuracies close to 1) and therefore difficult to improve. For large sample sizes ($N > 100$), performance were close (difference between mean accuracies lower than 0.6%) between models initialized with default algorithm and pre-trained models (see Figures 6 and 7). However, for smaller sample sizes (N=50), pre-training improved classification – similarly to what had been shown for more complex tasks – with accuracies of the pre-trained models higher than default models of 0.7% and 1.2% for task classification and one contrast task classification respectively. These results suggest that pre-training can be beneficial when studying difficult classification problems such as those with few training samples or complex classification tasks.

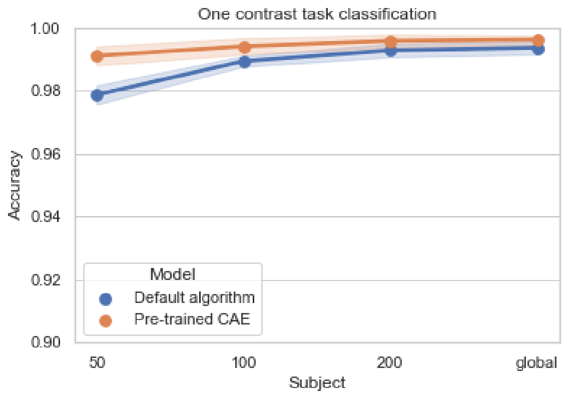

Figure 7: Mean accuracies and standard errors of the mean on one contrast task classification with the HCP dataset for the models initialized with default algorithm (blue) and pre-trained CAE (orange). Pre-training does not always improve one-contrast task classification performance: for large sample sizes, pre-training and default initialization give very similar results.

3.4 Benefits of self-taught learning on a heterogeneous dataset

Table 5 summarizes the results for the classification of mental concepts on the small and the large BrainPedia datasets. These results are illustrated in Figure 8.

Table 5: Classification performances on BrainPedia datasets of models initialized with default algorithm vs with the weights of a pre-trained CAE. DA: Default Algorithm initialization ; PT: Pre-Training initialization

**Dataset**	**Small BrainPedia**		**BrainPedia**	
**Init.**	**DA**	**PT**	**DA**	**PT**
**Mean acc. (%)**	56.8	64.5	67.1	74.2
(std. err.)	(1.5)	(2.1)	(0.9)	(2.3)
Paired T-test (*4 dof*)		**-8.72**		**-3.43**
p-value		***0.001***		***0.02***
**Mean F1-score (%)**	50.5	62.0	64.9	73.6
(std. err.)	(3.5)	(2.1)	(0.8)	(2.2)
Paired T-test (*4 dof*)		**-4.89**		**-2.89**
p-value		***0.008***		***0.04***

On a the small BrainPedia dataset, pre-training improved the performance of the classifier. When looking at the mean accuracies, respectively 56.8% and 64.5% for the classifier initialized with the default algorithm and the pre-trained classifier, the difference was high (almost 8% of improvement). But in this case, the F1-score is a better metric to assess the performance. Indeed, this metric focuses more on classification errors and is a better indicator of performance when classes are imbalanced, which is the case in this dataset in which some classes are more represented than others (*e.g.* in the small BrainPedia training set, 205 maps correspond to the class "visual words, language, visual" whereas only 19 are in the class "left foot, visual"). When focusing on this metric, the pre-trained classifier performance were markedly higher than the ones of the classifier with default initialization (11.5% of improvement in mean F1-score). Performance (accuracies and F1-scores) were both significantly improved with the pre-trained model compared (P-value < 0.05).

On the global BrainPedia dataset, performance also increased with pre-training. Mean accuracy and F1-score were higher for the the pre-trained model (F1-score of 73.6% against 64.9% for the model with default initialization) even if

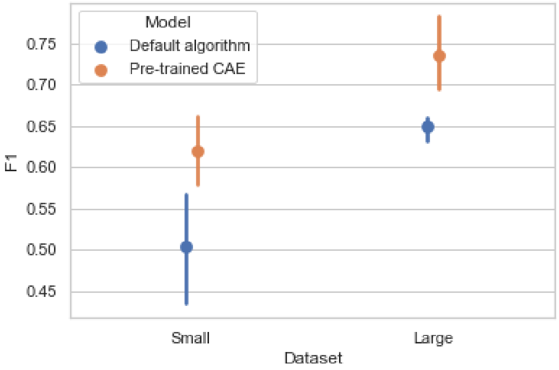

Figure 8: Mean F1-scores and standard errors of the mean of the classification of mental concepts on BrainPedia datasets (Small and Large) for the models initialized with default algorithm (blue) and pre-trained CAE (orange). Pre-training improves classification performance, in particular for the small dataset.

the sample size of the dataset was higher and more classes were represented. Indeed, the classification task was also more complex for this dataset since data were separated into 36 classes instead of 30 for Small BrainPedia due to the presence of maps from other studies in the dataset.

4 Discussion

4.1 Summary of the results

In this work, we showed the benefits of self-taught learning on two large public datasets with different sample sizes and classification tasks. In all cases, pre-training a classifier with an unsupervised task (in our case: reconstruction) was beneficial but the level of improvement varied depending on the classification task and the size of the training dataset.

When sample sizes were small, pre-training always improved the classification performance, regardless of whether the dataset was homogeneous or heterogeneous and of the complexity of the classification task. In medical imaging, where the dimensions of the data are often very large and few samples are typically available due to high financial and human costs, learning a good representation of the data can be very difficult [12, 51]. Unsupervised pre-training is thus helpful by initializing the weights of the CNN to preserve the (brain) structure learned by the autoencoder, and facilitate the learning process. However, when the sample size increased, benefits were less remarkable since the amount of available training data was probably sufficient to learn a good representation.

This observation can also be made for classification tasks. When trying to classify the data in a small number of classes, performance of the pre-trained classifier were better but not with a high improvement of performance, even for small sample sizes (*e.g.* 100 subjects for task classification). But when trying to separate data into more classes, for a more fine-grained classification, the representation learned during the pre-training was beneficial.

Another benefit of self-taught learning we found was the reduction of the training time. Performance of the pre-trained classifier were better even with less training epochs. This was the case for both datasets results which were computed for 500 epochs for the default algorithm and 200 epochs for the pre-trained model. This is in line with [52] in which researchers showed that the pre-trained models remain in the same basin of the loss function when trained on new data and since the weights are already initialized close to a good representation of data, less epochs are necessary to adapt this representation for classification.

Architectures of the models also had an impact on the benefits of self-taught learning. With both datasets, pre-trained models performed better using the 5-layers architecture. This effect was studied by [36] who showed that, while unsupervised pre-training helps for deep networks with more layers, it appears to hurt for too small networks. The size of the latent space of the CAE with 5-layers being almost 5 times smaller that the 4-layers one, it suggests that only a small subset of features of the input are relevant for predicting the class label.

However, the classification accuracies of the pre-trained models were not related to the reconstruction performance of the CAE since the 4-layers CAE reconstructs maps with better precision than the 5-layers CAE. This confirms that the

features learned by the 4-layers CAE for reconstruction were not all useful for classification and focusing on a smaller number of features (with 5-layers) facilitates the learning process.

4.2 Limitations and perpectives

Due to the high computational time required to train a model, we only compared two model architectures (4 and 5-layers). Other types of architectures with different number of fully-connected or convolutional layers could have been tested to see the effect of other latent space sizes as it was done in [36]. We also chose to evaluate the impact of whole transfer learning (*i.e.* transferring the weights of all convolutional layers) but it could be interesting to see if the effect is the same when transferring only the weights of the first layers, to see which part of the compressed data are the most impactful.

The main limitation of our work is the classification experiments and datasets we chose. In fMRI, the number of possible labels and thus, classification tasks is very high due to a lack of consensus in the field with respect to standardizing tasks, contrasts and mental concepts [33]. In our experiments, we used the labels provided by NeuroVault as specified in the original studies [16, 41]. We chose to compare multiple types of classification on the HCP dataset to illustrate different approaches are in use in the field or that were used by other studies [24, 23]. For BrainPedia, a multi-label decoding was performed in the original study since multiple concepts are associated with most maps. Labels we had access to were then the list of labels associated with each map. To be able to compare our results with those of the homogeneous dataset (HCP), we chose to classify these as unique labels, which is less complex and less precise in practice. This type of issue is due to the lack of harmonization in the way tasks and cognitive processes are defined. Using ontologies such as Cognitive Atlas [33], NeuroVault annotations could be harmonized and enriched, as it was done by [32] by mapping the original labels to target ones from Cognitive Atlas or [53] in which cognitive conditions were annotated by a group of expert using the same atlas.

In neuroimaging, many sources of variability can impact the results of an experiment and the generalizability of the results. Here, we investigated the generalizability of our model by assessing the benefits of pre-training on a heterogeneous dataset (BrainPedia). While this dataset was heterogeneous in terms of the studies that were included, all maps were obtained using the same processing pipeline. Multiple studies have shown that the exact pipeline used to obtain an fMRI result can have a non-negligeable impact on fMRI statistic maps [54, 55]. In the future, using a more variable dataset with statistic map from different studies but also processed using different pipelines would be of great interest. In a recent study [10], the authors tried to compare the performance of different classifiers trained on fMRI 3D volumes series obtained with various scenarios of minimal preprocessing pipelines. A similar experiment was recently made by [56] who found that preprocessing pipeline selection can impact the performance of a supervised classifier. Comparing the adaptation capacities of models on volumes preprocessed with different pipelines could be also interesting to evaluate the impact of analytical variability on deep learning with fMRI.

In this context, using unsupervised models could allow us to build a space capturing the similarities and differences of statistic maps, *i.e.* to learn a robust latent representation of the important features of statistic maps in a specific context. By adding other constraints to this latent space and/or choosing an adapted pre-training dataset, we could use this for other purposes than brain decoding. For instance, building a space that captures the analytical variability in statistic maps could help us understand the difference between the pipelines but also identify the more robust pipelines. Future works will focus on building such a space with specific constraints to evaluate distance between different pipelines.

5 Conclusion

In this study, we compared the benefits of a self-taught learning framework in the task of classifying 3D fMRI statistic maps. We showed that unsupervised pre-training improves the performance of classification in experiments with few training samples and complex classification tasks, which is a very common setup in fMRI studies.

6 Acknowledgements

This work was partially funded by Region Bretagne (ARED MAPIS) and Agence Nationale pour la Recherche for the programm of doctoral contracts in artificial intelligence (project ANR-20-THIA-0018). We thank Gregory Kiar who worked on a preliminary version of the autoencoder based on NeuroVault data. Data used in the HCP dataset were provided [in part] by the Human Connectome Project, WU-Minn Consortium (Principal Investigators: David Van Essen and Kamil Ugurbil; 1U54MH091657) funded by the 16 NIH Institutes and Centers that support the NIH Blueprint for Neuroscience Research; and by the McDonnell Center for Systems Neuroscience at Washington University.

7 Availability of data and materials

7.1 Data availability

The data used in this study are openly available on NeuroVault: `https://www.neurovault.org` [31]. No experimental activity involving the human participants was made by the authors. Only publicly released data were used.

NeuroVault IDs of statistic maps included in each dataset (NeuroVault, HCP and BrainPedia) are available in Derived data, see 7.3.

7.2 Code

The code produced to run the experiments and to create the figures and tables of this paper is available in the Software Heritage public archive at: `https://archive.softwareheritage.org/browse/origin/https://gitlab.inria.fr/egermani/self_taught_decoding`.

7.3 Derived data

Derived data such as NeuroVault IDs of statistic maps used in the different datasets and the parameters of the trained models (for validation of hyperparameter and test of performance) are available in Zenodo: `https://doi.org/10.5281/zenodo.7070890` [38].

7.4 Ethics

The data used in this study are openly available on NeuroVault: `https://www.neurovault.org` [31]. No experimental activity involving the human participants was made by the authors. Only publicly released data were used.

HCP: Written informed consent was obtained from all participants of HCP and the original study was approved by the Washington University Institutional Review Board.

BrainPedia: BrainPedia database is comprised of data from 29 studies, assembled from various sources (OpenNeuro, NeuroSpin research center, etc.). Subject-level maps resulting from first-level statistical analysis were upladed on NeuroVault by [41]. The list of the original dataset used is available on [41], S1 Text (`https://doi.org/10.1371/journal.pcbi.1006565.s001`).

References

[1] Anees Abrol, Zening Fu, Mustafa Salman, Rogers Silva, Yuhui Du, Sergey Plis, and Vince Calhoun. Deep learning encodes robust discriminative neuroimaging representations to outperform standard machine learning. *Nature Communications*, 2021.

[2] Wutao Yin, Longhai Li, and Fang-Xiang Wu. Deep learning for brain disorder diagnosis based on fMRI images. *Neurocomputing*, 469:332–345, 2022.

[3] Orhan Firat, Like Oztekin, and Fatos T. Yarman Vural. Deep learning for brain decoding. In *2014 IEEE International Conference on Image Processing (ICIP)*, pages 2784–2788. IEEE, 2014.

[4] Hanh Vu, Hyun-Chul Kim, and Jong-Hwan Lee. 3d convolutional neural network for feature extraction and classification of fMRI volumes. In *2018 International Workshop on Pattern Recognition in Neuroimaging (PRNI)*, pages 1–4, 2018.

[5] Jinlong Hu, Yuezhen Kuang, Bin Liao, Lijie Cao, Shoubin Dong, and Ping Li. A multichannel 2d convolutional neural network model for task-evoked fMRI data classification. *Computational Intelligence and Neuroscience*, 2019.

[6] Muhammad Bilal Qureshi, Laraib Azad, Muhammad Shuaib Qureshi, Sheraz Aslam, Ayman Aljarbouh, and Muhammad Fayaz. Brain decoding using fMRI images for multiple subjects through deep learning. *Computational and Mathematical Methods in Medicine*, 2022.

[7] Sotetsu Koyamada, Yumi Shikauchi, Ken Nakae, Masanori Koyama, and Shin Ishii. Deep learning of fmri big data: a novel approach to subject-transfer decoding. *arXiv preprint arXiv: 1502.00093*, 2015.

[8] Xiaoxiao Wang, Xiao Liang, Zhoufan Jiang, Benedictor A. Nguchu, Yawen Zhou, Yanming Wang, Huijuan Wang, Yu Li, Yuying Zhu, Feng Wu, Jia-Hong Gao, and Bensheng Qiu. Decoding and mapping task states of the human brain via deep learning. *Human Brain Mapping*, 2020.

[9] Xiaojie Huang, Jun Xiao, and Chao Wu. Design of deep learning model for task-evoked fMRI data classification. *Computational Intelligence and Neuroscience*, 2021.

[10] Hanh Vu, Hyun-Chul Kim, Minyoung Jung, and Jong-Hwan Lee. fMRI volume classification using a 3d convolutional neural network robust to shifted and scaled neuronal activations. *NeuroImage*, 2020.

[11] Kanghan Oh, Woosung Kim, Guangfan Shen, Yanhong Piao, Nam-In Kang, Il-Seok Oh, and Young Chul Chung. Classification of schizophrenia and normal controls using 3d convolutional neural network and outcome visualization. *Schizophrenia Research*, 212:186–195, 2019.

[12] Armin W. Thomas, Christopher Ré, and Russell A. Poldrack. Challenges for cognitive decoding using deep learning methods. *preprint arXiv:2108.06896*, 2021.

[13] Junghwan Cho, Kyewook Lee, Ellie Shin, Garry Choy, and Synho Do. How much data is needed to train a medical image deep learning system to achieve necessary high accuracy? *arXiv preprint arXiv:1511.06348*, 2015.

[14] Russell A. Poldrack, Chris I. Baker, Joke Durnez, Krzysztof J. Gorgolewski, Paul M. Matthews, Marcus R. Munafò, Thomas E. Nichols, Jean-Baptiste Poline, Edward Vul, and Tal Yarkoni. Scanning the horizon: towards transparent and reproducible neuroimaging research. *Nature Reviews Neuroscience*, 2017.

[15] Katherine S. Button, John P. A. Ioannidis, Claire Mokrysz, Brian A. Nosek, Jonathan Flint, Emma S. J. Robinson, and Marcus R. Munafò. Power failure: why small sample size undermines the reliability of neuroscience. *Nature Reviews Neuroscience*, 14, 2013.

[16] David C. Van Essen, Stephen M. Smith, Deanna M. Barch, Timothy E.J. Behrens, Essa Yacoub, and Kamil Ugurbil. The WU-minn human connectome project: An overview. *Mapping the Connectome*, 2013.

[17] Myriam Bontonou, Giulia Lioi, Nicolas Farrugia, and Vincent Gripon. Few-shot decoding of brain activation maps. *2021 29th European Signal Processing Conference (EUSIPCO)*, 2021.

[18] Sunao Yotsutsuji, Miaomei Lei, and Hiroyuki Akama. Evaluation of task fMRI decoding with deep learning on a small sample dataset. *Frontiers in neuroinformatics*, 2021.

[19] Peiye Zhuang, Alexander G Schwing, and Oluwasanmi Koyejo. Fmri data augmentation via synthesis. In *2019 IEEE 16th International Symposium on Biomedical Imaging (ISBI 2019)*, pages 1783–1787. IEEE, 2019.

[20] Arthur Mensch, Julien Mairal, Bertrand Thirion, and Gaël Varoquaux. Extracting representations of cognition across neuroimaging studies improves brain decoding. *PLOS Computational Biology*, 2014.

[21] Sinno Jialin Pan and Qiang Yang. A survey on transfer learning. *IEEE Transactions on Knowledge and Data Engineering*, 2010.

[22] Jia Deng, Wei Dong, Richard Socher, Li-Jia Li, Kai Li, and Li Fei-Fei. ImageNet: A large-scale hierarchical image database. In *2009 IEEE Conference on Computer Vision and Pattern Recognition*, pages 248–255, 2009.

[23] Armin W. Thomas, Ulman Lindenberger, Wojciech Samek, and Klaus-Robert Müller. Evaluating deep transfer learning for whole-brain cognitive decoding. *arXiv preprint arXiv: 2111.01562*, 2021.

[24] Yufei Gao, Yameng Zhang, Hailing Wang, Xiaojuan Guo, and Jiacai Zhang. Decoding behavior tasks from brain activity using deep transfer learning. *IEEE Access*, 2019.

[25] Michele Svanera, Mattia Savardi, Sergio Benini, Alberto Signoroni, Gal Raz, Talma Hendler, Lars Muckli, Rainer Goebel, and Giancarlo Valente. Transfer learning of deep neural network representations for fMRI decoding. *Journal of Neuroscience Methods*, 2019.

[26] Nahiyan Malik and Danilo Bzdok. From youtube to the brain: Transfer learning can improve brain-imaging predictions with deep learning. *Neural Networks*, 153:325–338, 2022.

[27] Will Kay, Joao Carreira, Karen Simonyan, Brian Zhang, Chloe Hillier, Sudheendra Vijayanarasimhan, Fabio Viola, Tim Green, Trevor Back, Paul Natsev, et al. The kinetics human action video dataset. *arXiv preprint arXiv:1705.06950*, 2017.

[28] Armin W Thomas, Christopher Ré, and Russell A Poldrack. Self-supervised learning of brain dynamics from broad neuroimaging data. *arXiv preprint arXiv:2206.11417*, 2022.

[29] Russell A Poldrack and Krzysztof J Gorgolewski. Making big data open: data sharing in neuroimaging. *Nature Neuroscience*, 2014.

[30] Christopher J Markiewicz, Krzysztof J Gorgolewski, Franklin Feingold, Ross Blair, Yaroslav O Halchenko, Eric Miller, Nell Hardcastle, Joe Wexler, Oscar Esteban, Mathias Goncavles, Anita Jwa, and Russell Poldrack. The OpenNeuro resource for sharing of neuroscience data. *eLife*, 2021.

[31] Krzysztof J. Gorgolewski, Gael Varoquaux, Gabriel Rivera, Yannick Schwarz, Satrajit S. Ghosh, Camille Maumet, Vanessa V. Sochat, Thomas E. Nichols, Russell A. Poldrack, Jean-Baptiste Poline, Tal Yarkoni, and Daniel S. Margulies. NeuroVault.org: a web-based repository for collecting and sharing unthresholded statistical maps of the human brain. *Frontiers in Neuroinformatics*, 2015.

[32] Romuald Menuet, Raphael Meudec, Jérôme Dockès, Gael Varoquaux, and Bertrand Thirion. Comprehensive decoding mental processes from web repositories of functional brain images. *Scientific Reports*, 2022.

[33] Russell A. Poldrack, Aniket Kittur, Donald Kalar, Eric Miller, Christian Seppa, Yolanda Gil, D. Stott Parker, Fred W. Sabb, and Robert M. Bilder. The cognitive atlas: Toward a knowledge foundation for cognitive neuroscience. *Frontiers in Neuroinformatics*, 2011.

[34] Rajat Raina, Alexis Battle, Honglak Lee, Benjamin Packer, and Andrew Y. Ng. Self-taught learning: transfer learning from unlabeled data. In *Proceedings of the 24th international conference on Machine learning*, pages 759–766. ACM Press, 2007.

[35] Hua Wang, Feiping Nie, and Heng Huang. Robust and discriminative self-taught learning. *Proceedings of the 30th International Conference on Machine Learning*, 2013.

[36] Dumitru Erhan, Yoshua Bengio, Aaron Courville, Pierre-Antoine Manzagol, Pascal Vincent, and Samy Bengio. Why does unsupervised pre-training help deep learning? *The Journal of Machine Learning Research*, 2010.

[37] Seyedmehdi Orouji, Vincent Taschereau-Dumouchel, Aurelio Cortese, Brian Odegaard, Cody Cushing, Mouslim Cherkaoui, Mitsuo Kawato, Hakwan Lau, and Megan AK Peters. " task-relevant autoencoding" enhances machine learning for human neuroscience. *arXiv preprint arXiv:2208.08478*, 2022.

[38] Elodie Germani, Elisa Fromont, and Camille Maumet. On the benefits of self-taught learning for brain decoding - data, 2022.

[39] Collection n°4337. NeuroVault Collection n°4337. `https://identifiers.org/neurovault.collection:4337`. Accessed: 2022-01-19.

[40] Collection n°1952. NeuroVault Collection n°1952. `https://identifiers.org/neurovault.collection:1952`. Accessed: 2022-01-19.

[41] Gaël Varoquaux, Yannick Schwartz, Russell A. Poldrack, Baptiste Gauthier, Danilo Bzdok, Jean-Baptiste Poline, and Bertrand Thirion. Atlases of cognition with large-scale human brain mapping. *PLOS Computational Biology*, 2018.

[42] Alexandre Abraham, Fabian Pedregosa, Michael Eickenberg, Philippe Gervais, Andreas Mueller, Jean Kossaifi, Alexandre Gramfort, Bertrand Thirion, and Gael Varoquaux. Machine learning for neuroimaging with scikit-learn. *Frontiers in Neuroinformatics*, 2014.

[43] Adam Paszke, Sam Gross, Francisco Massa, Adam Lerer, James Bradbury, Gregory Chanan, Trevor Killeen, Zeming Lin, Natalia Gimelshein, Luca Antiga, Alban Desmaison, Andreas Kopf, Edward Yang, Zachary DeVito, Martin Raison, Alykhan Tejani, Sasank Chilamkurthy, Benoit Steiner, Lu Fang, Junjie Bai, and Soumith Chintala. Pytorch: An imperative style, high-performance deep learning library. In *Advances in Neural Information Processing Systems 32*, pages 8024–8035. 2019.

[44] Shane Cook. *CUDA Programming: A Developer's Guide to Parallel Computing with GPUs*. 2012.

[45] Sergey Ioffe and Christian Szegedy. Batch normalization: Accelerating deep network training by reducing internal covariate shift. *CoRR*, abs/1502.03167, 2015.

[46] Diederik P Kingma and Jimmy Ba. Adam: A method for stochastic optimization. *arXiv preprint arXiv:1412.6980*, 2014.

[47] Charles R. Harris, K. Jarrod Millman, Stéfan J. van der Walt, Ralf Gommers, Pauli Virtanen, David Cournapeau, Eric Wieser, Julian Taylor, Sebastian Berg, Nathaniel J. Smith, Robert Kern, Matti Picus, Stephan Hoyer, Marten H. van Kerkwijk, Matthew Brett, Allan Haldane, Jaime Fernández del Río, Mark Wiebe, Pearu Peterson, Pierre Gérard-Marchant, Kevin Sheppard, Tyler Reddy, Warren Weckesser, Hameer Abbasi, Christoph Gohlke, and Travis E. Oliphant. Array programming with NumPy. *Nature*, 2020.

[48] Kaiming He, Xiangyu Zhang, Shaoqing Ren, and Jian Sun. Delving deep into rectifiers: Surpassing human-level performance on imagenet classification. In *Proceedings of the IEEE international conference on computer vision*, pages 1026–1034, 2015.

[49] Gaël Varoquaux and Veronika Cheplygina. Machine learning for medical imaging: methodological failures and recommendations for the future. *npj Digital Medicine*, 2022.

[50] Sayash Kapoor and Arvind Narayanan. Leakage and the reproducibility crisis in ml-based science. *arXiv preprint arXiv:2207.07048*, 2022.

[51] Chen Sun, Abhinav Shrivastava, Saurabh Singh, and Abhinav Gupta. Revisiting unreasonable effectiveness of data in deep learning era. In *Proceedings of the IEEE International Conference on Computer Vision (ICCV)*, pages 843–852, 2017.

[52] Behnam Neyshabur, Hanie Sedghi, and Chiyuan Zhang. What is being transferred in transfer learning? *Advances in neural information processing systems*, 33:512–523, 2020.

[53] Jonathon Walters, Maedbh King, Patrick G. Bissett, Richard B. Ivry, Jörn Diedrichsen, and Russell A. Poldrack. Predicting brain activation maps for arbitrary tasks with cognitive encoding models. *NeuroImage*, 263:119610, 2022.

[54] Joshua Carp. On the plurality of (methodological) worlds: estimating the analytic flexibility of fMRI experiments. *Frontiers in Neuroscience*.

[55] Rotem Botvinik-Nezer, Felix Holzmeister, Colin F Camerer, Anna Dreber, Juergen Huber, Magnus Johannesson, Michael Kirchler, Roni Iwanir, Jeanette A Mumford, R Alison Adcock, et al. Variability in the analysis of a single neuroimaging dataset by many teams. *Nature*, 582(7810):84–88, 2020.

[56] Xinhui Li, Alex Fedorov, Mrinal Mathur, Anees Abrol, Gregory Kiar, Sergey Plis, and Vince Calhoun. Pipeline-invariant representation learning for neuroimaging. *arXiv preprint arXiv:2208.12909*, 2022.

Click here to access/download
Supplementary Material
supplementary_figure_S1.pdf

Click here to access/download
Supplementary Material
supplementary_table_S1.pdf

Click here to access/download
Supplementary Material
supplementary_table_S2.pdf

Click here to access/download
Supplementary Material
supplementary_table_S3.pdf

Click here to access/download
Supplementary Material
supplementary_table_S4.pdf

Click here to access/download
Supplementary Material
supplementary_table_S5.pdf

Click here to access/download
Supplementary Material
supplementary_table_S6.pdf

Cover letter
Click here to access/download;Personal Cover;Cover-letter-Gigascience-Germani.pdf

October 10th, 2022
To: Scott Edmunds, PhD
Editor-in-Chief, GigaScience

Dear Professor Edmunds,

We are pleased to submit our manuscript, “On the benefits of self-taught learning for brain decoding”, for consideration for publication in GigaScience.

Deep learning approaches are increasingly used in the field of brain imaging. Yet, the typical size of neuroimaging studies associated with the large number of features in each brain image makes it particularly challenging to extract meaningful latent representations of the brain. Here, we took advantage of NeuroVault — a large open database of fMRI statistic maps — in a self-taught learning framework. We pre-trained an unsupervised deep learning model (autoencoder) to learn a latent representation of fMRI statistic maps on the full NeuroVault database (28,532 maps) and we fine-tuned this model for multiple decoding tasks. We tested our classifier on two large collections of the NeuroVault database: a homogeneous collection of more than 18000 statistic maps (from the HCP) and a heterogeneous collection of 6500 maps (BrainPedia).

We found that unsupervised pre-training improves the performance of classification in experiments with few training samples (50 to 100 subjects) and with complex classification tasks (classifying contrasts), a setup that is often found in neuroimaging studies. We believe that our methodology and trained model could systematically help the development of new brain decoding methods.

We have made publicly available all the code to reproduce our analyses and the figures.

Thank you for considering our manuscript. We would be very happy to provide any additional information that you may need.

Yours Sincerely,

Camille Maumet, Elisa Fromont and Elodie Germani

RENNES – BRETAGNE ATLANTIQUE
Campus universitaire de Beaulieu
35042 Rennes Cedex – France
Phone: +33 (0)2 99 84 71 00

www.inria.fr
