# Supplementary material for: On the benefits of self-taught learning for brain decoding
Source: Gigascience. 2023 May 3;12:giad029. doi: 10.1093/gigascience/giad029 (PMC10155221; doi:10.1093/gigascience/giad029)

Supplementary Figure S1 - Schematic visualisation of the architectures of the Convolutional AutoEncoder (a) and Convolutional Neural Network (b) with 4 layers

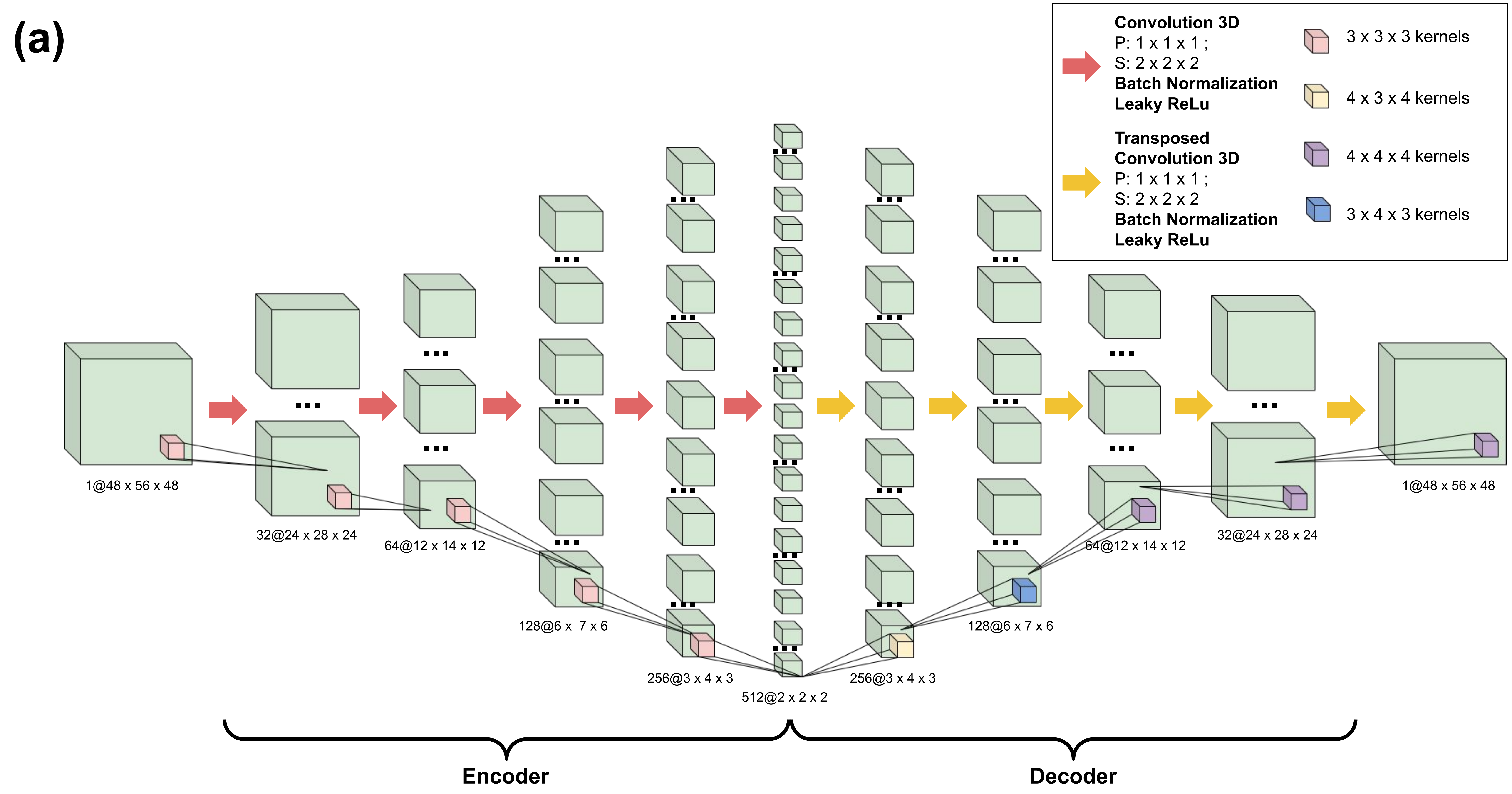

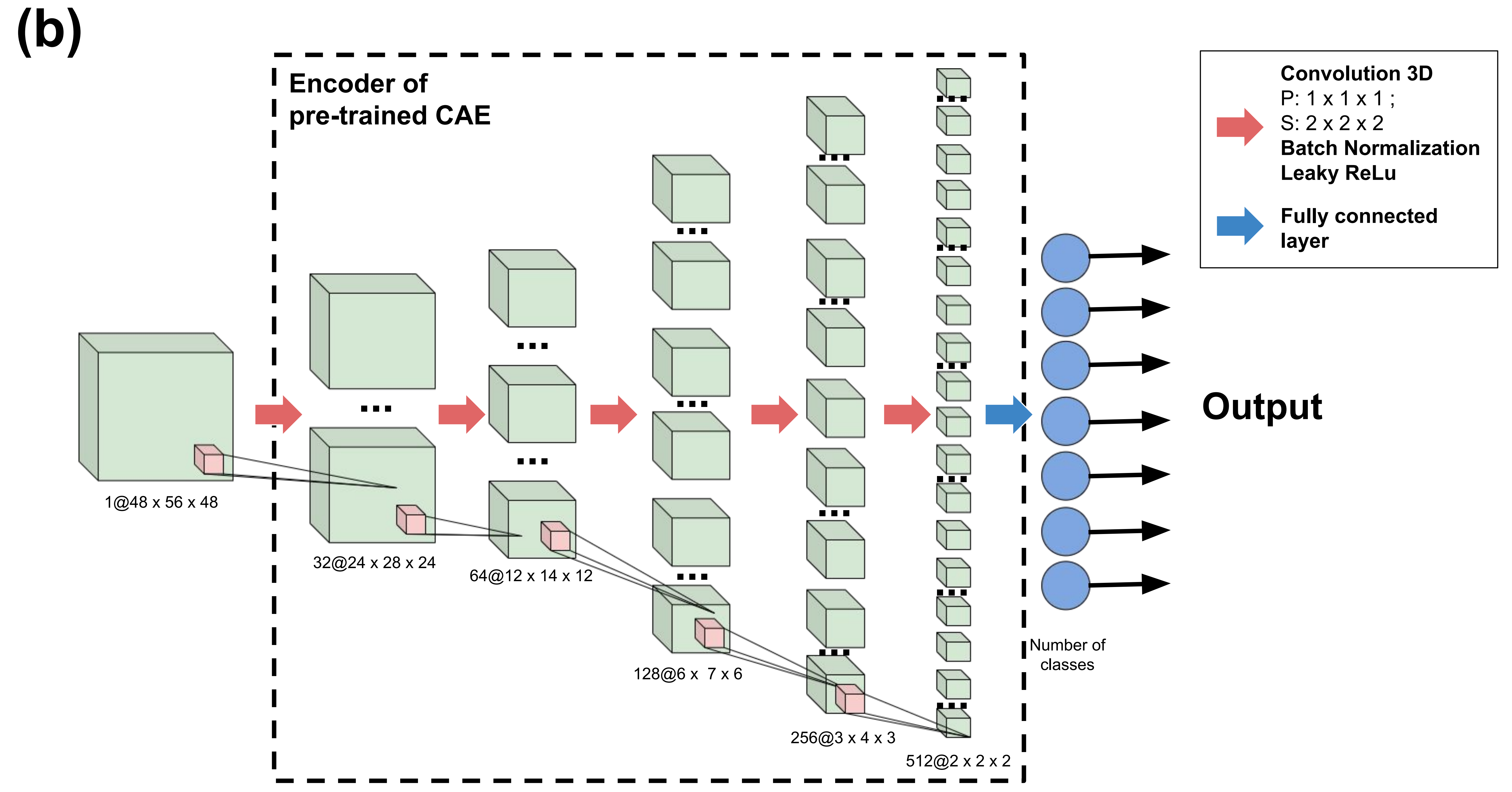

Supplement: giad029_Supplemental_Files [file giad029_supplemental_files.zip › supplementary_figure_S1.pdf]
