# Supplementary material for: On the benefits of self-taught learning for brain decoding
Source: Gigascience. 2023 May 3;12:giad029. doi: 10.1093/gigascience/giad029 (PMC10155221; doi:10.1093/gigascience/giad029)
Supplement: giad029_Supplemental_Files [file giad029_supplemental_files.zip › supplementary_figure_S2.pdf]

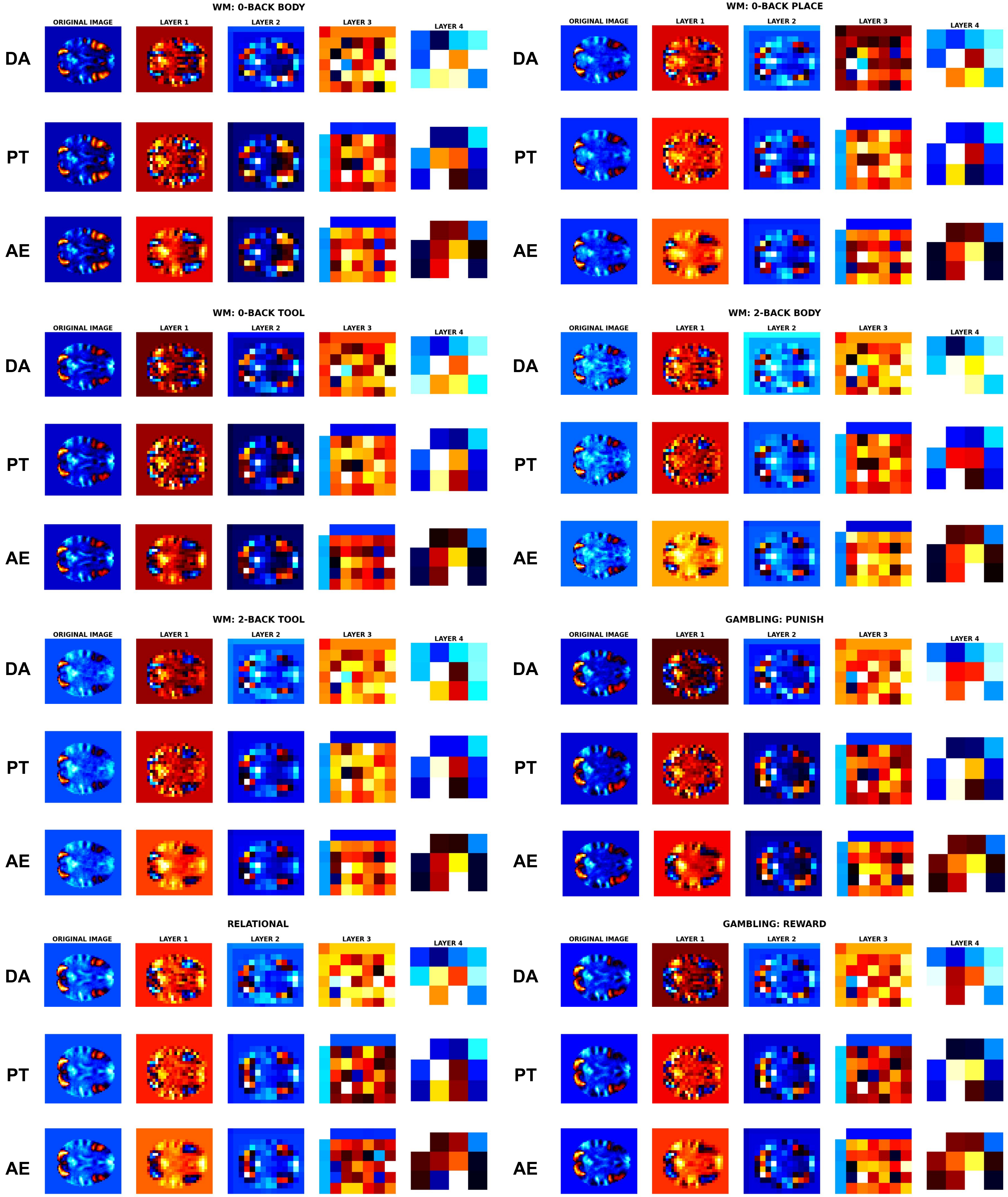

Supplementary Figure S2. Original mean statistic maps (column 1) and mean feature maps across subjects of the fold 1 of the test dataset of HCP 50 for the first four convolutional layers of each model (columns 2-4): CNN with default algorithm initialization (DA), pre-trained CNN (PT) and CAE for the 8 selected contrasts: `Working Memory': `0-back body', `0-back places', `0-back tools', `2-back body', `2-back tools' , `Gambling: punish', `Gambling: reward and `Relational.
