# Supplementary material for: On the benefits of self-taught learning for brain decoding
Source: Gigascience. 2023 May 3;12:giad029. doi: 10.1093/gigascience/giad029 (PMC10155221; doi:10.1093/gigascience/giad029)
Supplement: giad029_Response_to_Reviewer_Comments_Original_Submission [file giad029_response_to_reviewer_comments_original_submission.pdf]

# Author's Response To Reviewer Comments

Close

Dear Editor-in-Chief, Co-editors, Reviewers,
We would like to thank the reviewers for their thoughtful comments. You may find a new uploaded document named "answers_to_reviewers.pdf", which provides answers to the reviewers issues and details on the updates performed in order to comply with the reviewer's comments. In this document, the reviewer's remarks appear in red bold face, our answers are in plain text and updates made to the manuscript are in italic and highlighted in yellow. Modifications are also highlighted in yellow in the updated manuscript.

Close
