# Supplementary material for: On the benefits of self-taught learning for brain decoding
Source: Gigascience. 2023 May 3;12:giad029. doi: 10.1093/gigascience/giad029 (PMC10155221; doi:10.1093/gigascience/giad029)
Supplement: giad029_Response_to_Reviewer_Comments_Revision_1 [file giad029_response_to_reviewer_comments_revision_1.pdf]

Author's Response To Reviewer Comments

Close

Dear Editor-in-chief, Editors, Reviewers,

We are pleased to submit the revision of our manuscript, “On the benefits of self-taught learning for brain decoding”, for consideration for publication in GigaScience.

We prepared the final version of the manuscript taking into account the suggestion of the reviewer, in particular:
- We simplified Figures 1 and 2 (for instance, as suggested by the reviewer, we removed the description of the cross-validation in Figure 1);
- We included a new table (Table 6) in the manuscript and a supplementary table (S7) which contains the per-class accuracy used to select contrasts in Section 3.5.1;
- We added a line in Table 7 of the updated manuscript to facilitate comparison of models;
- We took in consideration the suggestion of the reviewer to add other reconstruction metrics (such as the mean square error) to evaluate the autoencoder performance and decided not to add these due to the difficulty to interpret these metrics;
- We added information about the number of subjects in the global HCP dataset on Table 4 and Figure 6, 7 and 8;
- We carefully read the two references suggested by the reviewer but decided not to include them in the manuscript. We have provided a full rationale for this in the attached response.

A complete point-by-point reply to the reviewer's comments is provided along with this new revision.

We hope that these modifications will help improve the quality of our final manuscript.
Thank you for considering our manuscript. We would be very happy to provide any additional information that you may need.

Close
