# Supplementary material for: On the benefits of self-taught learning for brain decoding
Source: Gigascience. 2023 May 3;12:giad029. doi: 10.1093/gigascience/giad029 (PMC10155221; doi:10.1093/gigascience/giad029)
Supplement: giad029_Response_to_Reviewer_Comments_Revision_2 [file giad029_response_to_reviewer_comments_revision_2.pdf]

Author's Response To Reviewer Comments

Close

Dear Editor,
Thank you for your answer. We made the modifications mentioned in the decision letter regarding the references to the GigaDB dataset and other URL mentioned in our original manuscript. We also changed the Abstract with a structure. Finally, we provide you the TeX file of the manuscript along with the PDF version.
Looking forward to hear from you for the production stage.
Best regards,
Elodie Germani, Elisa Fromont and Camille Maumet

Close
