# Supplementary material for: On the benefits of self-taught learning for brain decoding
Source: Gigascience. 2023 May 3;12:giad029. doi: 10.1093/gigascience/giad029 (PMC10155221; doi:10.1093/gigascience/giad029)
Supplement: giad029_Reviewer_1_Report_Original_Submission — Huiguang He -- 11/2/2022 Reviewed [file giad029_reviewer_1_report_original_submission.pdf]

Reviewer Report

Title: On the benefits of self-taught learning for brain decoding

Version: Original Submission **Date:** 11/2/2022

Reviewer name: Huiguang He

Reviewer Comments to Author:

The authors proposed a self-taught algorithm that using large-scale neural datasets to train a CAE and consequently using the trained encoder to initialize a supervised convolutional neural network to classify tasks or cognitive processes of unseen statistic maps. The proposed method is compared with the default initialization method on a homogenous dataset and a heterogeneous dataset. The results indicate that the self-taught learning process always improves the performance of the classifiers. The pre-training can be more beneficial when studying more difficult classification problems such as those with few training samples or complex classification tasks. The authors also found that pre-training can reduce training time and achieve model convergence in a shorter number of iterations.

Pros

1. The authors make the data and code available. It is helpful for other researchers to reproduce the work.
2. The paper is well-written. Descriptions of the most of experimental settings are clear. The authors conducted adequate experiments.

Cons

1. The method is not novel. The idea that using the model weights of the encoder of a pre-trained autoencoder to initialize a classification network is a very common application in deep learning community.
2. The contributions are trivial. The authors focus on the performance of the self-taught method on different datasets, different sample sizes, and different classification tasks. However, more profound analyses related to neural signals and computational neurosciences are needed. For example, the authors can compare the similarities and differences in statistic map representations on the hidden space learned by CAE and the hidden space learned by the classifier (by default initialization or the pre-trained encoder).

Some questions:

1. Is the number of statistic maps the same as the number of samples?
2. In Section 2.5.2, is the t-test used in the experiment a one-tailed or two-tailed t-test?
3. In Section 2.6.1, the authors said that they selected a single contrast per task and classified the tasks (7 different tasks, with one contrast per task). On what principle was the selection made? Or was it a random selection?
4. In Section 4.2, the authors said that the high computational time is required to train the CAE model. How long does it take to train a CAE model?
5. In the process of training the classifier, how are the results if the weights of the first stage pre-trained encoder are fixed and only the fully connected layer is trained? I also suggest that the authors

complement the experiments to explore the effect of transferring the weights of part of CAE layers on the results.

Methods

Are the methods appropriate to the aims of the study, are they well described, and are necessary controls included? Choose an item.

Conclusions

Are the conclusions adequately supported by the data shown? Choose an item.

Reporting Standards

Does the manuscript adhere to the journal's guidelines on [minimum standards of reporting?]() Choose an item.

Choose an item.

Statistics

Are you able to assess all statistics in the manuscript, including the appropriateness of statistical tests used? Choose an item.

Quality of Written English

Please indicate the quality of language in the manuscript: Choose an item.

Declaration of Competing Interests

Please complete a declaration of competing interests, considering the following questions:

- Have you in the past five years received reimbursements, fees, funding, or salary from an organisation that may in any way gain or lose financially from the publication of this manuscript, either now or in the future?
- Do you hold any stocks or shares in an organisation that may in any way gain or lose financially from the publication of this manuscript, either now or in the future?
- Do you hold or are you currently applying for any patents relating to the content of the manuscript?
- Have you received reimbursements, fees, funding, or salary from an organization that holds or has applied for patents relating to the content of the manuscript?
- Do you have any other financial competing interests?
- Do you have any non-financial competing interests in relation to this paper?

If you can answer no to all of the above, write 'I declare that I have no competing interests' below. If your reply is yes to any, please give details below.

I declare that I have no competing interests

I agree to the open peer review policy of the journal. I understand that my name will be included on my report to the authors and, if the manuscript is accepted for publication, my named report including any attachments I upload will be posted on the website along with the authors' responses. I agree for my report to be made available under an Open Access Creative Commons CC-BY license (http://creativecommons.org/licenses/by/4.0/). I understand that any comments which I do not wish to be included in my named report can be included as confidential comments to the editors, which will not be published.

Choose an item.

To further support our reviewers, we have joined with Publons, where you can gain additional credit to further highlight your hard work (see: https://publons.com/journal/530/gigascience). On publication of this paper, your review will be automatically added to Publons, you can then choose whether or not to claim your Publons credit. I understand this statement.

Yes Choose an item.
