# Supplementary material for: On the benefits of self-taught learning for brain decoding
Source: Gigascience. 2023 May 3;12:giad029. doi: 10.1093/gigascience/giad029 (PMC10155221; doi:10.1093/gigascience/giad029)
Supplement: giad029_Reviewer_1_Report_Revision_1 — Huiguang He -- 2/2/2023 Reviewed [file giad029_reviewer_1_report_revision_1.pdf]

Reviewer Report

Title: On the benefits of self-taught learning for brain decoding

Version: Revision 1 **Date:** 2/2/2023

Reviewer name: Huiguang He

Reviewer Comments to Author:

The authors provide a comprehensive response to my earlier questions and supplement the advised experiments. However, I still have some questions and suggestions.
Major:
1. Table 2 only uses Pearson correlation to quantify the reconstruction performance of the CAE. Previous studies usually use multiple image-similarity metrics to assess image reconstruction performance. I suggest the authors use other metrics, such as MSE (mean-square error) and SSIM (similarity structural index measure) for a more comprehensive evaluation.
2. I suggest the author plots Figure 1 more concisely. The exact process of 5-fold cross-validation can be omitted as cross-validation is a very common operation in machine learning and is not the focus of Figure 1. Figure 2 has a similar problem. There are too many cuboids in Figure 2. It is not necessary since the texts like 128@12x14x12 in Figure 2 already present the specific parameters of the network structure.
3. In the revised paper, the authors 'focused on the contrasts that led to the most difficult classification tasks (i.e had the lowest per-class accuracy (less than 80%))' when visualizing the features in Section 3.5.1. However, I didn't find the corresponding tables or figures showing the per-class accuracy. The authors should provide the results of per-class accuracy.
4. In Figure 10, there is a large difference between the correlation of PT and AE in Layer 4. For DA and AE, the correlation increases with deeper layers. However, for PT, the correlation in Layer 4 is lower than that in Layer 3 (0.9 -> 0.8). How to explain them?
5. The case where the number of transferred layers equals zeroï¼Œi.e., the DA, should be listed in Table 6 for comparison. In Table 7, the condition where Nb. Of frozen layers equals to one is missing.
6. In Section 3.5.2, the authors claim that the results in Table 6 'suggest that features learned by the CAE at this layer were less important for classification'. I don't agree with the authors. I think the results can only suggest that only transferring features learned by the CAE at the first layer were insufficient for classification. Insufficient does not mean less important. The authors' conclusion can only be drawn if the model that transfers only the first layer weights of CAE does not work as well as the model trained with random initialization.
Minor:
1. It would be better to indicate how many subjects are included in the global dataset in Table 4 and at the horizontal axes of Figure 6 and Figure 7.
2. There are several recent studies on brain decoding based on fMRI. The authors can cite them in the Introduction.
[1] Du, Changde, et al. "Structured neural decoding with multitask transfer learning of deep neural

network representations." IEEE Transactions on Neural Networks and Learning Systems 33.2 2022, doi: 10.1109/TNNLS.2020.3028167.
[2] Kim J-H, Zhang Y, Han K, Wen Z, Choi M, Liu Z., "Representation learning of resting state fMRI with variational auto-encoder," NeuroImage, 241: 118423, 2021
Writing issues:
1. Please revise the following sentences. These seem to have grammatical errors that make them difficult to understand.
Page 10: In the second paragraph of Section 2.7.2, 'transferring only the weights of one single up to the first four first convolutional layers'. In the third paragraph of Section 2.7.2, 'freezing of two to five first convolutional layers'.
2. Typos:
Page 10: In the second paragraph of Section 2.7.2, 'transfered' -> 'transferred'.
The caption of Figure 9: '(columns 2-4)' -> '(columns 2-5)'
Page 17: In the first paragraph of Section 3.5.1: 'Gambling: reward and 'Relational -> 'Gambling: reward' and 'Relational: relational'?

Methods

Are the methods appropriate to the aims of the study, are they well described, and are necessary controls included? Choose an item.

Conclusions

Are the conclusions adequately supported by the data shown? Choose an item.

Reporting Standards

Does the manuscript adhere to the journal's guidelines on minimum standards of reporting? Choose an item.

Choose an item.

Statistics

Are you able to assess all statistics in the manuscript, including the appropriateness of statistical tests used? Choose an item.

Quality of Written English

Please indicate the quality of language in the manuscript: Choose an item.

Declaration of Competing Interests

Please complete a declaration of competing interests, considering the following questions:

- Have you in the past five years received reimbursements, fees, funding, or salary from an organisation that may in any way gain or lose financially from the publication of this manuscript, either now or in the future?
- Do you hold any stocks or shares in an organisation that may in any way gain or lose financially from the publication of this manuscript, either now or in the future?
- Do you hold or are you currently applying for any patents relating to the content of the manuscript?
- Have you received reimbursements, fees, funding, or salary from an organization that holds or has applied for patents relating to the content of the manuscript?
- Do you have any other financial competing interests?
- Do you have any non-financial competing interests in relation to this paper?

If you can answer no to all of the above, write 'I declare that I have no competing interests' below. If your reply is yes to any, please give details below.

I declare that I have no competing interests

I agree to the open peer review policy of the journal. I understand that my name will be included on my report to the authors and, if the manuscript is accepted for publication, my named report including any attachments I upload will be posted on the website along with the authors' responses. I agree for my report to be made available under an Open Access Creative Commons CC-BY license (http://creativecommons.org/licenses/by/4.0/). I understand that any comments which I do not wish to be included in my named report can be included as confidential comments to the editors, which will not be published.

Choose an item.

To further support our reviewers, we have joined with Publons, where you can gain additional credit to further highlight your hard work (see: https://publons.com/journal/530/gigascience). On publication of this paper, your review will be automatically added to Publons, you can then choose whether or not to claim your Publons credit. I understand this statement.

Yes Choose an item.
